# *Igf2* adult-specific skeletal muscle enhancer activity revealed in mice with intergenic CTCF boundary deletion

**DOI:** 10.1371/journal.pgen.1011834

**Published:** 2025-08-29

**Authors:** Joanne L. Thorvaldsen, Aimee M. Juan, Yemin Lan, Christopher Krapp, Marisa S. Bartolomei

**Affiliations:** University of Pennsylvania Perelman School of Medicine, Epigenetics Institute, Department of Cell and Developmental Biology, Philadelphia, Pennsylvania, United States of America; University of Melbourne, AUSTRALIA

## Abstract

Precise, monoallelic expression of imprinted genes is governed by *cis* regulatory elements called imprinting control regions (ICRs) and enhancer-promoter (E-P) interactions shaped by local chromatin architecture. The *Igf2/H19* locus employs allele-specific CTCF binding at the ICR to instruct enhancer accessibility to maternal *H19* and paternal *Igf2* promoters. Here, we investigate the CTCF-bound centrally conserved domain (CCD), intergenic to *H19* and *Igf2*, and an adjacent widely expressed lncRNA. Using transgenic mice, deletion alleles reinforced CCD as a neonatal muscle-specific repressor of maternal *Igf2*. However, deletion of the abutting lncRNA did not affect *Igf2* levels. Unexpectedly, in adult skeletal muscle where *Igf2* is normally repressed, absence of CCD resulted in remarkable, high-level activation of *Igf2* from both parental alleles. Through multimodal chromatin analyses, we identified a conserved putative adult skeletal muscle enhancer (PaSME) insulated between chromatin domains at ICR and CCD. We propose that removal of CCD allows PaSME to drive robust abnormal *Igf2* activation on both alleles in adult skeletal muscle. Thus, we uncover CCD as a developmental biallelic muscle-specific repressor, adding a new layer of architectural regulation to the extensively studied *Igf2/H19* locus.

## Author summary

All mammals have a unique class of genes that are crucial for normal growth, known as “imprinted genes,” where only one gene copy inherited from a parent is active. Imprinted genes are usually expressed in specific cell types and at certain stages of development, but the instructions for how this pattern is achieved is not fully understood. Our study uses the well-known imprinted *Igf2/H19* locus as a model for studying complex gene regulation, as *Igf2* plays important roles in muscle cell growth. Between the two genes lies a genetic boundary called the Centrally Conserved Domain (CCD) and a proposed activating sequence that we called the putative adult skeletal muscle enhancer (PaSME). Using transgenic mice and advanced DNA folding analyses, we show that CCD acts as a blocker of PaSME. This barrier keeps *Igf2* expression appropriately off in adult muscle. When CCD is deleted, this barrier is lost, allowing PaSME to activate *Igf2* to abnormally highly levels in muscle, leading to increased body weight in some animals. This mechanism enhances our understanding of how imprinted genes are controlled at critical timepoints, and their impact on development and disease.

## Introduction

Imprinted genes comprise a small but developmentally important set of genes in mammals that are expressed from a single parental allele. Imprinted gene regulation requires *cis* elements, including imprinting control regions (ICRs) and enhancers, to achieve allele and tissue specific expression essential for normal development [1]. At the imprinted murine *Igf2*/*H19* locus, the paternally-expressed insulin growth factor 2 (*Igf2*) gene and the maternally-expressed long non-coding RNA (lncRNA)-encoding *H19* gene are regulated by the intergenic 2 kb ICR (2kb upstream of *H19*) in combination with enhancers downstream of *H19* and *Igf2*. The mouse and human *IGF2*/*H19* loci share spatial and sequence conservation of regulatory elements [2]. The ICR serves as a DNA methylation-sensitive CTCF boundary, mutations and epimutations of which are associated with imprinting disorders Beckwith-Wiedemann syndrome and Silver-Russell syndrome [2,3]. Although tissue-specific enhancers for embryonic and neonatal liver and skeletal muscle have been defined [4–6], the ubiquitous expression patterns of *Igf2* and *H19* across development require additional undefined *cis*-acting sequences.

Imprinted *Igf2* and *H19* levels increase throughout preimplantation development in all tissues and are repressed postnatally, with a few exceptions. For example, in adult skeletal muscle, *H19* expression persists while *Igf2* is repressed [7–9]. In adult brain, *Igf2* persists and is biallelically expressed in specific tissues such as choroid plexus and leptomeninges, or is maternally-expressed in a subset of neurons [7,10–14]. Together, these exceptions indicate that distinct regulatory mechanisms direct *H19* and *Igf2* expression in brain and adult skeletal muscle.

To address other potential regulatory elements that govern tissue and developmental-specific expression, we and others examined the 130-kb region spanning the *Igf2*/*H19* locus *in vivo*. A conserved 2kb GC-rich region was identified between *H19* and *Igf2* that is hypomethylated and contains prominent and ubiquitous DNase I hypersensitive sites (DHS) [15]. This region, coined the centrally conserved domain (CCD) [16], is composed of two elements with high identity to human sequence [17]. Unlike the maternally-bound ICR, CTCF and cohesin were detected on both alleles at CCD [18]. Furthermore, transgenic and deletion studies evaluating CCD indicated that it may function as an *Igf2* brain-specific enhancer and/or a skeletal muscle-specific repressor of the maternal *Igf2* allele, likely through CTCF-mediated looping [16,17,19,20].

Adjacent to CCD lies a lncRNA (designated intervening region or *Ivr*) identified in embryonic and neonatal tissues with hallmarks common to most lncRNAs (RNA polymerase II transcribed, polyadenylated and spliced) [21,22]. Transcription of *Ivr* was reported to be in the direction of and anti-sense to *Igf2*, but the role of *Ivr* at the imprinted *H19/Igf2* locus is unknown. Given the coincident expression of *Ivr* and *Igf2* in early development, and the proximity of *Ivr* to CCD, we hypothesized that *Ivr* may contribute to CCD-directed tissue-specific regulation of *Igf2*.

Here we further analyze lncRNA *Ivr* and its association with CCD-dependent *Igf2* expression at different stages in development. First, we demonstrate that *Ivr* is preferentially expressed from the paternal allele and its paternal-specific expression is dependent upon the *Igf2*/*H19* ICR in multiple tissues. Next, by deleting *Ivr*, we show that it is not required for CCD-dependent repression of maternal *Igf2* in skeletal muscle. Co-deletion of *Ivr* and CCD recapitulates reported skeletal muscle-specific activation of maternal *Igf2* in neonates [17,19]. Moreover, in adults, deletion of CCD and *Ivr* from either allele results in high level *Igf2* expression in skeletal muscle. Our 3D chromatin analyses in skeletal muscle, together with publicly available 2D chromatin data, indicate the CCD interaction with sequence proximal to *Igf2* serves as a boundary to adult-specific skeletal muscle enhancer activity. The biological function of *Ivr* remains to be elucidated.

## Results

### Characterization of the intervening region *(Ivr)* transcript adjacent to the *Igf2*/*H19* intergenic CCD-hypersensitivity site

**Fig 1:**
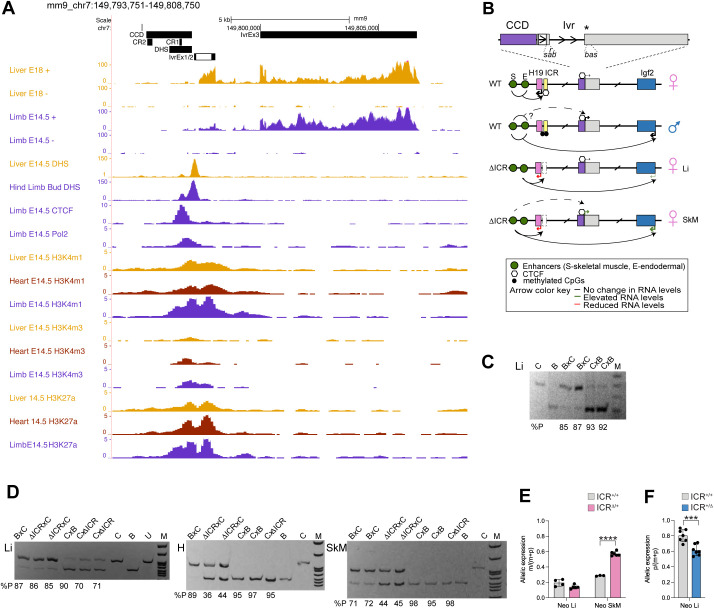
Analysis of *Igf2*/*H19* intervening lncRNA *Ivr* and CCD. (A) UCSC genome browser view of annotated CCD and *Ivr* regions using publicly available ENCODE data, including tissue-specific RNA-seq, DNAse HS-seq and ChIP-seq for relevant protein and histone marks (Sheet A in S2 Table). (B) Schematic for CCD (purple box)/*Ivr* (gray boxes) with relative locations of primers used to identify transcription start sites (TSSs) and splice junctions (r, s*),* primers used for allelic expression (*a*, *b*) and SNP (*) (Sheet C in S3 Table). *Avr*II digestion discriminates the C57BL/6 (B) and *Mus Castaneus* (C) *Ivr* transcripts. Allele-specific expression for *H19* (pink box), *Igf2* (blue box) and *Ivr* are depicted for WT alleles and *ΔICR* alleles. Tissue-specific enhancers (green circles) regulate maternal *H19* and paternal *Igf2* (curved arrows) and may regulate paternal *Ivr* (dashed arrow). CTCF bound ICR (yellow box) blocks enhancers from engaging maternal *Igf2*. (C) Allele-specific *Ivr* in WT (BxC and CxB) neonatal liver (Li). (D) Allele-specific *Ivr* from WT and *ΔICR* alleles in neonatal Li, heart (H) and skeletal muscle (SkM). Primer pairs ‘*a*’ and ‘*b*’ in (B) were used in (C) and (D), respectively. For (C) and (D), genotype and ladder lane M (BR322 DNA-*Msp*I) are above panels; % expression from paternal allele [%p = 100xp/(p + m)] of *Avr*II digested qRT-PCR DNA are noted below panels. U corresponds to undigested PCR product. (E) Maternal WT versus *ΔICR Ivr* expression [m/(m + p)] in neonatal (Neo) Li and SkM. (F) Paternal WT versus *ΔICR* I*vr* expression [p/(m + p)] in Neo Li. (Two-tailed Welch’s T-test, ***p < 0.001; ****p < 0.0001; error bars represent SD).

To identify additional regulatory elements that may contribute to tissue- and allele-specific *Igf2* expression, we examined the transcript (*Ivr*) adjacent to CCD, which was first recognized in a mouse imprinting region tiling array (MIRTA) that described lncRNAs associated with imprinted loci [22]. *Ivr* was characterized as a spliced, ~ 8kb transcript expressed in embryonic and neonatal tissues (Fig 1A annotation and 1B schematic). Two *Ivr* transcription start sites (TSSs) and Exon1/2 splice junctions were identified from alignment of cloned 5’RACE DNA (Sheet A in S1 Table) [22]. Embryonic samples from the Mouse ENCODE project showed transcription coincident with *Ivr* in the direction of and anti-sense to *Igf2* (Fig 1A and Sheet A in S2 Table). Publicly available DHS and ChIP-seq data from multiple embryonic tissues indicated that the *Ivr* TSSs are adjacent to the DHS and CTCF binding sites of CCD. Additionally, the *Ivr* promoter is positioned within open chromatin decorated with signatures of promoters as well as enhancers (Pol2, H3K4me1, H3K4me3 and H3K27ac) (Fig 1A and Sheet A in S2 Table) [23–25].

We evaluated the allele-specific expression of *Ivr* because it was intervening to imprinted genes *H19* and *Igf2*. Using RT-PCR with primers in Exons 2 and 3, we first confirmed expression of *Ivr* in neonatal tissues and sequenced Exon 2/3 splice junction from cDNA (Fig 1B, 1C, and 1D and Sheet B in S1 Table). Next, in progeny from reciprocal matings between C57BL/6 (B) and Cast7 (C) mice, which have a *Mus castaneus* chromosome 7 on a B background, we evaluated *Ivr* allelic expression by restriction fragment length polymorphism (RFLP). Coincident with its location on the *Igf2* side of the ICR, *Ivr* was preferentially expressed from the paternal allele in neonatal liver, skeletal muscle and heart (Fig 1B,1C,1D). Remarkably, inheriting the *Igf2*/**H19* ICR* deletion (*ΔICR*) [26] from the maternal allele resulted in higher maternal *Ivr* levels in skeletal muscle and heart but not liver (Fig 1A, 1D, 1E). However, on the paternal *ΔICR* allele, *Ivr* levels were reduced in liver (Fig 1D, 1F). These results follow the trend for *Igf2* tissue-specific activation on the *ΔICR* maternal allele (high in skeletal muscle and heart, low in liver) and repression on the *ΔICR* paternal allele (most repressed in liver) [26,27]. The tissue-specific *Igf2* levels from *ΔICR* alleles may be due to how each tissue-specific enhancer is shared by *H19* and *Igf2* on the *ΔICR* alleles. Our results suggest that *Ivr* expression is partly regulated by tissue-specific *Igf2* shared enhancers downstream of *H19* on the paternal allele that are blocked by ICR insulator activity on the maternal allele. While the skeletal muscle enhancer (SME) and endodermal enhancer (EE) for liver have been identified, heart specific enhancer remains undefined (S1 Fig genome browser annotation) [4,5].

### Generation and analysis of *ΔIvr* and *ΔCCDIvr* alleles

As stated above, transgenic and deletion studies evaluating CCD indicated that it may be a skeletal muscle-specific repressor of the maternal *Igf2* allele [17,19]. To test the hypothesis that *Ivr* is required for CCD to repress muscle-specific *Igf2* expression, we derived several deletion alleles in mice. We generated an allele with *Ivr* deleted and CCD floxed (S2A Fig). Mice with the targeted *Δ*Ivr + Neo** allele were mated with Flp mice for removal of NeoR cassette to generate the *Δ*Ivr** allele. These offspring were then mated with Cre mice to generate mice with the *ΔCCDIvr* allele. Alleles were confirmed by Southern blot and PCR analyses (S2A and S2B Fig and Sheets A and B in S3 Table). The *Δ*Ivr** and *ΔCCDIvr* mice were mated with the Cast7 mice to generate progeny for *Igf2* allelic expression.

### *Igf2* is derepressed from the maternal *ΔCCDIvr* but not the *ΔIvr* allele in neonatal skeletal muscle

**Fig 2:**
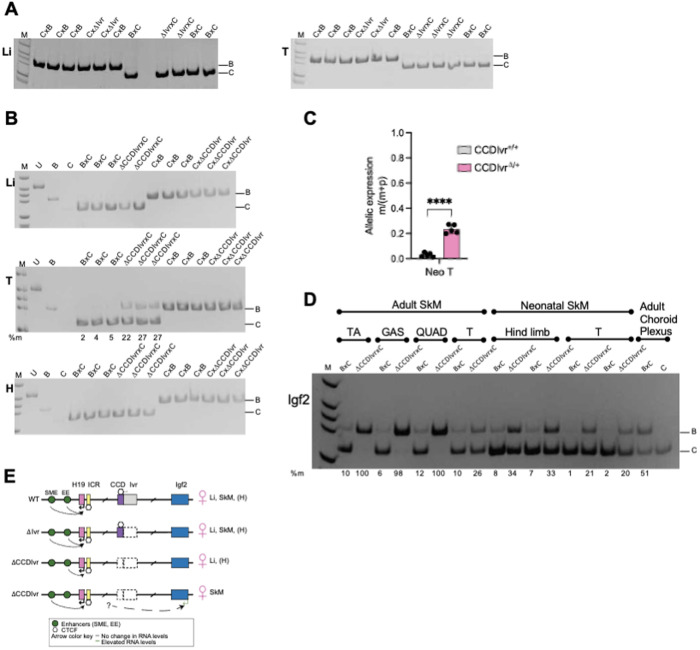
*Igf2* activation from the maternal Δ*CCDIvr* allele in skeletal muscle. (A) Allele-specific *Igf2* expression in liver (Li) and tongue (T) from neonates inheriting WT and *Δ*Ivr** allele paternally (CxB and Cx*Δ*Ivr**) and maternally (BxC and *Δ*Ivr**xC). For (A-D), *Igf2* qRT-PCR products were digested with *MluC*I to discriminate the parental B (WT B, *Δ*Ivr**, *ΔCCDIvr*) and C alleles. In (B), allele-specific *Igf2* expression in Li, T and heart (H) from neonates inheriting WT and *ΔCCDIvr* allele maternally (BxC and *ΔCCDIvr*xC) and paternally (CxB and CxΔ*CCDIvr*). In (B) (T panel) and (D), *Igf2* expression from maternal *ΔCCDIvr* allele is noted below the gel. (C) *Igf2* expression from maternal WT versus *ΔCCDIvr* allele in neonatal T (Two-tailed Welch’s T-test, ****p < 0.0001; error bars represent SD.) (D) Maternal-specific *Igf2* expression in adult TA, GAS, QUAD, T and choroid plexus, and neonatal skeletal muscle (hindlimb and T) WT (BxC) and mutant *(ΔCCDIvr*xC) tissues as indicated above gel. For panels (A), (B) and (D): M (ladder), U (undigested), B and C (control tissues), %m (%maternal = 100 x m/(m + p). (E) Schematic of *H19*, *Ivr* and *Igf2* expression from WT, *Δ*Ivr** and *ΔCCDIvr* maternal alleles. *Igf2* is expressed from the maternal *ΔCCDIvr* allele in neonatal SkM.

In neonates inheriting the maternal *Δ*Ivr** allele, maternal *Igf2* was not activated (Fig 2A), demonstrating that the *Ivr* alone does not repress maternal *Igf2*. As expected, neonates inheriting the maternal *ΔCCDIvr* allele exhibited maternal *Igf2* activation exclusively in neonatal skeletal muscle (tongue, Fig 2B and 2C) [17,19]. In contrast, *Igf2* levels were unchanged in neonates inheriting the paternal *ΔCCDIvr* allele (Fig 2B; S2C Fig). Furthermore, *H19* was unaffected regardless of the transmission of the *ΔCCDIvr* alleles (S2D, S2E Fig). Finally, we detected similar levels of *Igf2* activation from the maternal *ΔCCDIvr* allele in neonatal skeletal muscle from tongue and hindlimb (Fig 2B, 2D). Our results for the *ΔCCDIvr* allele are consistent with the previous reports that (1) deleted the conserved CCD DHS sequence (leaving *Ivr* intact) from a 130-kb imprinted *Igf2*-*H19* transgene [17] and (2) deleted the endogenous CCD together with *Ivr* sequence [19].Together, these data indicate that CCD is an *Igf2* skeletal muscle-specific repressor on the maternal allele (Fig 2E). Despite the critical role of *Igf2* in early growth, inheritance of the *ΔCCDIvr* alleles did not affect total body weight of neonates (S2F Fig), although there was a trend in weight gain in adult *CCDIvr*^*Δ/+*^ and *CCDIvr*^*+/Δ*^ mice relative to wild-type (WT) littermates (S3A Fig). Because adult-specific weight gain was previously reported in mice expressing high levels of *Igf2* in adult tissues [28], we next tested whether the observed trend in adult weight gain in *ΔCCDIvr* mice was associated with derepression of *Igf2* in skeletal muscle*.*

### *Igf2* is highly activated from both parental *ΔCCDIvr* alleles in adult skeletal muscle

**Fig 3:**
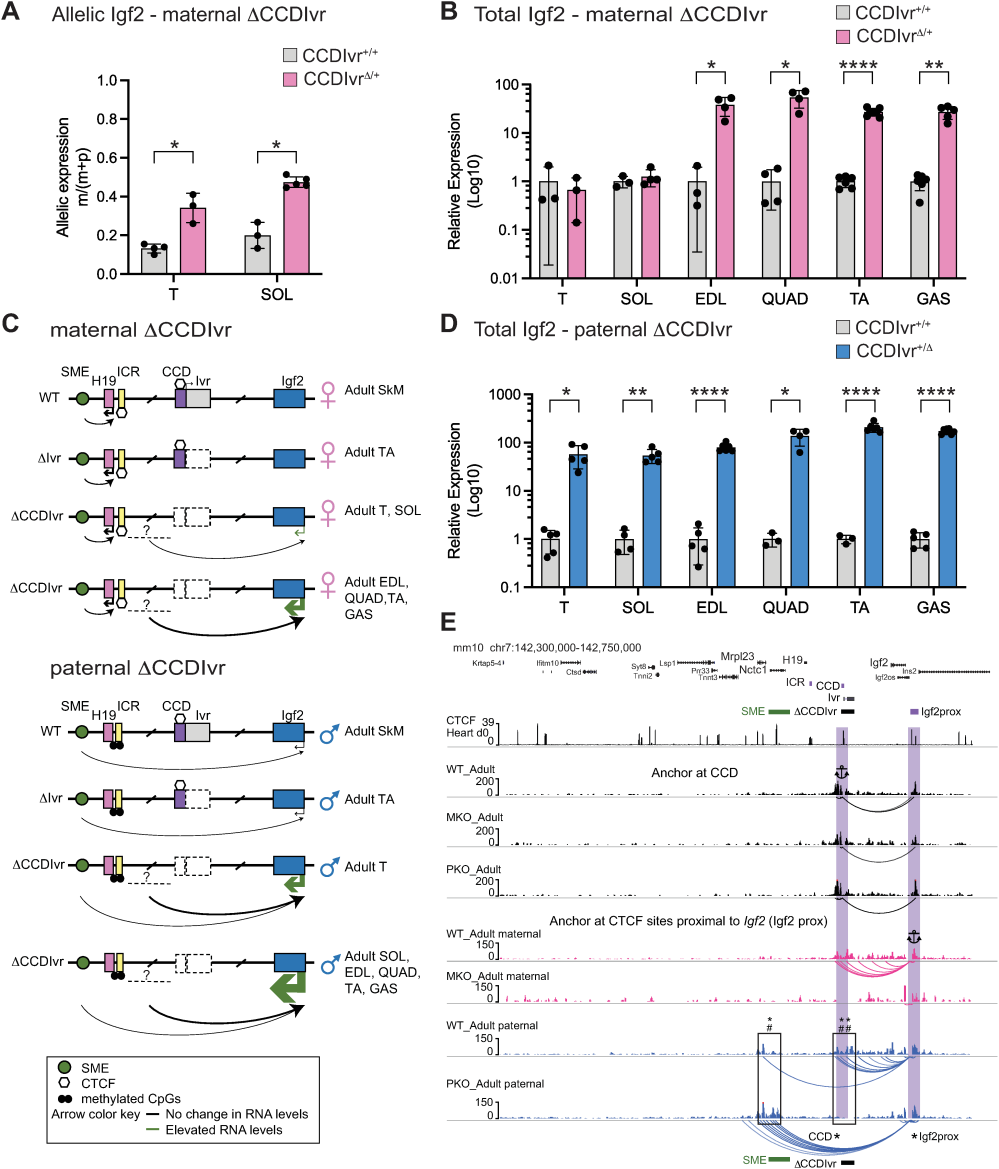
*Igf2* expression and Capture C interactions with proximal *Igf2* CTCF binding site in adult *ΔCCDIvr* skeletal muscle. (A) Maternal allele-specific *Igf2* expression in *CCDIvr*^*+/+*^ versus *CCDIvr*^*Δ/+*^ tongue (T) and soleus (SOL). (B) Total *Igf2* expression in *CCDIvr*^*+/+*^ versus *CCDIvr*^*Δ/+*^ T, SOL, EDL, QUAD, TA and GAS. (C) Schematic of *H19*, *Ivr* and *Igf2* expression from WT, *Δ*Ivr** and *ΔCCDIvr* maternal alleles (top) and paternal alleles (bottom) in adult skeletal muscle. (D) Total *Igf2* expression in *CCDIvr*^*+/+*^ versus *CCDIvr*^*+/Δ*^ T, SOL, EDL, QUAD, TA and GAS. For (B) and (D), relative *Igf2* expression in mutant vs WT muscle normalized to 1, depicted on log10 scale (y-axis) (Two-tailed Welch’s t-test: *p < 0.05, **p < 0.01, ***p < 0.001, ****p < 0.0001; error bars represent SD). (E) Normalized Capture C tracks from CTCF sites at CCD (Anchor at CCD, not allele-specific) and sequence proximal to *Igf2* (Anchor at Igf2prox, allele-specific) in adult skeletal muscle of WT, *CCDIvr*^*Δ/+*^ (MKO) and *CCDIvr*^*+/Δ*^ (PKO) mice. All data presented on WashU Epigenome Browser using mouse mm10 (Sheet A in S2 Table). Interactions at CCD anchor (black - both alleles) and at Igf2prox anchor (pink - maternal alleles, blue - paternal alleles), are highlighted (purple bars). For CCD Anchor, WT-Adult presents interactions for both parental WT alleles, MKO-Adult presents track from WT paternal allele and PKO-Adult presents track from WT maternal allele. From Igf2prox anchor, on WT-Adult paternal versus PKO_Adult paternal, boxed regions indicated gain and loss of interactions to *Mrpl23*/SME CTCF sites and CCD, respectively. Wald statistics using two methods to identify peaks are presented (* or # p < 0.05, ** or ## p < 0.01, Sheet E in S5 Table).

We first evaluated allele-specific *Igf2* expression by RFLP in tongue (T) and easily accessible larger hind limb muscles including gastrocnemius (GAS), quadriceps (QUAD) and tibialis anterior (TA) in adult mice inheriting the maternal *ΔCCDIvr* allele. Unexpectedly, we measured a large increase in maternal *Igf2* activation in the adult hind limb muscles compared to the adult T (Fig 2D). We reasoned that this difference was linked to the extensive transcriptional diversity across muscle subtypes [29]. We therefore extended our analysis to include hind limb muscles extensor digitorum (EDL) (with an overlapping transcriptional profile to GAS, QUAD and TA) and soleus (SOL) (with an overlapping transcriptional profile to T) to capture other possible skeletal muscle-specific outcomes with the genetic deletion of CCD*Ivr*. Accordingly, in adult *CCDIvr*^*Δ/+*^ mice, maternal *Igf2* was modestly activated in T and SOL (34% and 47% of total *Igf2*, respectively) (Fig 3A), similar to neonatal skeletal muscle (Fig 2B-2D). In stark contrast, maternal *Igf2* activation in EDL, QUAD, TA and GAS greatly exceeded expression from the paternal WT *Igf2* allele. Compared to WT, *Igf2* was elevated on average 38-, 54-, 27- and 27-fold in *CCDIvr*^*Δ/+*^ EDL, QUAD, TA and GAS, respectively (Fig 3B, 3C). These results suggest that maternal CCD*Ivr* functions as an insulator, deletion of which allows maternal *Igf2* access to robust skeletal muscle-specific enhancer activity. Note that as with neonatal tissues, *Igf2* activation was not dependent upon *Ivr* alone, because *Igf2* was not expressed from the maternal *Δ*Ivr** allele (*Ivr*^*-/+*^) in adult TA (S3B Fig).

We next examined *Igf2* on the paternal *ΔCCDIvr* allele in adult muscle tissues. Chromatin conformation capture (3C) in human culture cells indicated that hypomethylated CCD sequence interacted with CTCF-bound sequence upstream of *IGF2* on both alleles and included *IGF2* in a chromatin loop or topologically-associated domain (TAD) [18,30]. We anticipated that this CTCF-dependent architecture could be blocking *Igf2* from a regulatory sequence such as an enhancer in skeletal muscle. Thus, we hypothesized that for maternal and paternal *ΔCCDIvr* alleles, loss of CTCF anchor points in the sub-TAD would release the putative enhancer from insulation. This element would then be equally accessible to both parental *Igf2* promoters and lead to similar *Igf2* activation. Compared to WT mice, *Igf2* levels were elevated on average 57-, 54-, 79-, 135-, 206- and 173-fold in *CCDIvr*^*+/Δ*^ T, SOL, EDL, QUAD, TA and GAS, respectively (Fig 3C, 3D). Unexpectedly, these *Igf2* levels from the paternal *ΔCCDIvr* allele were much higher than *Igf2* activation from the maternal *ΔCCDIvr* allele (Fig 3A, 3B). This result was in stark contrast to *CCDIvr*^*+/Δ*^ neonatal T in which paternal *Igf2* expression was unaffected (Fig 2B; S2C Fig), supporting the role of a developmental and tissue-specific enhancer that is insulated by CCD. Furthermore, this enhancer activity was elevated by access to SME on the paternal *ΔCCDIvr* allele (Fig 3C, 3D).

Endodermal and skeletal muscle enhancers distal/centromeric to *H19* have been characterized for neonatal *H19* and *Igf2* expression [4,5]. On the paternal *ΔCCDIvr* allele, *Igf2* has access to skeletal muscle enhancer (SME) overlapping *Nctc1* (S1A Fig) [5,31]. SME, which is blocked by the ICR on maternal allele, regulates paternal-specific expression of *Igf2* and maternal specific expression of *H19* in skeletal muscle. At least two scenarios could explain high *Igf2* levels on the *ΔCCDIvr* alleles: (1) CCD normally blocks this SME from engaging *Igf2* promoters and deleting CCD increases its probability to engage these promoters or (2) a previously uncharacterized SME between the ICR and CCD was exposed with the CCD*Ivr* deletion (Fig 3C). To address these possibilities, we next investigated how loss of CCD-dependent architecture in neonatal and adult skeletal muscle tissues could account for elevated *Igf2* from *ΔCCDIvr* alleles*.*

### CCD interacts with CTCF-bound sequence upstream of *Igf2* in neonatal and adult skeletal muscle on both alleles

The region surrounding the *Igf2*/*H19* locus contains multiple CTCF binding sites that play a role in shaping local chromatin architecture. We first aimed to map the constitutive topologically-associated domains (TADs) within the *Igf2/H19* imprinted locus. To this end, we utilized published and publicly available ENCODE data across multiple cell-types to confirm local TAD boundaries and associated CTCF/cohesin binding sites surrounding *Igf2*, including CCD (S1 Fig and Sheet A in S2 Table). Once the bulk of the chromatin landscape was defined, we then genetically dissected allele-specific CCD-dependent chromatin architecture *in vivo* using WT and *ΔCCDIvr* deletion alleles in mice. Using a targeted chromatin conformation approach, we interrogated the local *Igf2/H19* gene architecture and reinforced the insulator role of CCD*Ivr* in neonatal and adult skeletal muscle tissues.

We first retrieved Micro-C data performed in embryonic stem cells (ESCs) [32] to confirm the constitutive local TAD that encompasses the *Igf2*/*H19* locus. We then aligned the TAD boundaries with CTCF (d0 Heart) and cohesin (CH12 cell line) ChIP sequencing data, as well as CTCF binding site motifs and motif orientation (S1 Fig and Sheets A and D in S2 Table). This analysis revealed multiple intra-TAD intersecting ‘dots’ between convergent CTCF sites (S1 Fig, centromeric green CTCF and telomeric red CTCF), indicating “pause sites” for loop extrusion [33–35]. ‘Dots’/loops between Igf2prox CTCF sites and multiple convergent CTCF sites across the locus are marked in S1 Fig (purple arrowheads), and include CTCF bound CCD (S1 Fig, dashed lines), supporting our hypothesis that CCD is contributing to local gene architecture. These findings were consistent with chromatin conformation analysis in human cell lines demonstrating that CCD interacts with CTCF-bound sequence proximal to *IGF2* (syntenic to Igf2prox, S1 Fig and Sheet D in S2 Table) on both parental alleles [30]. In mouse cell lines, this biparental CCD/*Igf2* interaction had not been demonstrated. Previous work using allele-specific CTCF-ChIP and 4C-seq chromatin conformation analysis in hybrid mouse ESCs mapped allele-specific architecture surrounding the ICR and Igf2prox CTCF binding sites at the *H19/Igf2* locus, but CCD was not addressed (S1 Fig) [34]. We also highlighted multiple looping/intra-TAD interactions from the Igf2prox CTCF sites, underscoring the need to consider additional *cis*-regulatory elements as important regulators of *Igf2*/*H19* expression (Micro-C in S1 Fig) [32–34,36].

To assess whether the previously characterized chromatin architecture in pluripotent cell line is preserved in *ΔCCDIvr* skeletal muscle tissues with elevated levels of *Igf2* expression, we conducted allelic chromatin conformation analyses in neonatal and adult F1 hybrid maternal and paternal *ΔCCDIvr* skeletal muscle nuclei (Sheets A-C in S4 Table). Using next-generation Capture-C technology, we designed probes to capture interactions from predicted looping sites across the TAD encompassing the *Igf2*/*H19* locus [37]. Where available, SNPs in the hybrid mice enabled discrimination of maternal versus paternal allele-captured interactions (S4 Table). We first examined allele-specific chromatin interactions on WT alleles in neonatal (WT-Neo) and adult (WT-Adult) skeletal muscle (S1 Fig, anchors at CCD [no SNP], Igf2prox [SNP], and ICR [SNP]) and confirmed known looping interactions of Igf2prox (paternal allele) and ICR (maternal allele) to convergent CTCF sites centromeric to *H19* in skeletal muscle, as demonstrated in ESCs [34] (S1 Fig, Igf2prox and ICR anchors). These looping interactions (S1 Fig, boxed region; Sheets A-D in S5 Table) bring the skeletal muscle enhancer (SME) [5] into proximity of *Igf2* on the paternal allele and *H19* on the maternal allele, facilitating allele-specific expression of each promoter in skeletal muscle. Next, we confirmed biallelic looping between CCD and Igf2prox in WT skeletal muscle, consistent with what was reported from syntenic regions in human cells (S1 Fig, purple bars, CCD anchor [non-allelic], Igf2prox anchor [bi-allelic]) [30]. To understand how CCD-dependent architecture may repress *Igf2* expression in adult skeletal muscle, we next examined the architectural impact of the CCD*Ivr* deletion.

### Elevated and altered looping interactions on *ΔCCDIvr* alleles in adult skeletal muscle

We investigated whether, in the absence of CCD, new looping interactions emerge from the Igf2prox-anchored region. Igf2prox anchored loops brought the SME and *Igf2* promoter into proximity on the paternal allele (S1 Fig). We hypothesized that this anchor could capture new enhancer-promoter (E-P) interactions on Δ*CCDIvr* alleles, which would explain elevated *Igf2* expression in adult skeletal muscle. First, CCD anchored looping to Igf2prox was observed from the WT alleles in both maternal and paternal *CCDIvr* deletion nuclei, indicating biallelic CCD-Igf2prox looping (Fig 3E, CCD anchor). As expected, on both maternal and paternal *ΔCCDIvr* alleles, Igf2prox looping to the deleted CCD*Ivr* region was absent. However, no new Igf2prox-anchored looping interactions were detected (S3C Fig, neonatal; Fig 3E, adult [boxed region at CCD]; Sheet E in S5 Table). Finally, in adult skeletal muscle, Igf2prox-anchored interaction peaks to CTCF sites near SME were higher on the paternal *ΔCCDIvr* allele compared to the corresponding paternal WT allele (Fig 3E, boxed region surrounding SME; Sheet E in S5 Table). This may occur because loss of the competing CCD/Igf2prox loop increases chances for SME/Igf2prox looping. The increased proximity of *Igf2* to SME (S3E Fig) likely contributes to elevated *Igf2* expression on the paternal *ΔCCDIvr* allele in adult skeletal muscle.

**Fig 4:**
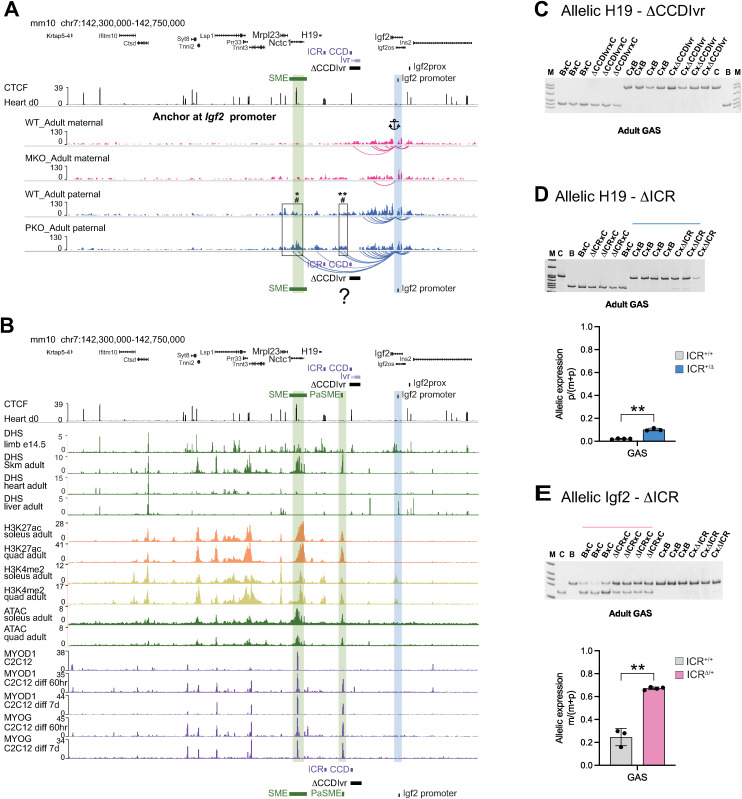
Evidence for a *Igf2* PaSME between the ICR and CCD in adult skeletal muscle. (A) Normalized allele-specific Capture-C peaks on WT, MKO and PKO alleles. Captured interactions from anchor at *Igf2* promoter (blue bar) reveals enhancer-promoter (E-P) interactions on the paternal PKO allele (Sheet F in S5 Table). (A) Boxed regions at SME and sequence between ICR and CCD (?) are significant E-P interactions present on the paternal PKO allele as measured by Wald statistics using two methods (* or # p < 0.05, ** p < 0.01, Sheet F in S5 Table). (B) Sequence between ICR and CCD with hallmarks of an adult SkM enhancer (PaSME) (Sheet A in S2 Table). (1) ENCODE DHS data show an adult SkM specific peak at PaSME (green bar). (2) Enhancer mark H3K27ac, H3K4me2 and ATAC peaks at PaSME in adult SkM. (3) ENCODE MYOD1 and MYOG ChIP-seq peak in differentiated myocytes coincide with PaSME. (C) Allele-specific *H19* expression from maternal and paternal WT versus *ΔCCDIvr* alleles in adult GAS. Allele-specific *H19* (D) and Igf2 (E) expression from maternal and paternal WT versus ΔICR alleles in adult GAS. Paternal-specific *H19* levels from WT versus paternal *ΔICR* allele (D) and maternal-specific *Igf2* levels from WT versus maternal *ΔICR* allele (E). For (D) and (E), two-tailed Welch’s t-test: **p < 0.01; error bars represent SD.

To directly measure *Igf2* E-P interactions, we next evaluated interactions captured from an *Igf2* promoter anchor probe (12 kb centromeric to the Igf2prox anchor [Sheet A in S4 Table]). This region was previously shown to loop to the SME on the paternal allele using chromatin conformation capture (3C) in neonatal skeletal muscle [31]. Consistently, the *Igf2* promoter anchor (blue bar) captured peaks at the SME (green bar) on the paternal but not the maternal WT alleles in skeletal muscle (S4A Fig [neonatal]; Fig 4A [adult]; Sheets F and G in S5 Table). *Igf2* promoter/SME looping was also captured on the paternal *ΔCCDIvr* alleles (Fig 4A; S4A Fig). In adult muscle, SME interaction peaks were higher on the paternal *ΔCCDIvr* versus WT allele (Fig 4A, boxed region; Sheet F in S5 Table), demonstrating that deleting CCD increases the probability of SME to engage *Igf2* promoters. Interestingly, a new *Igf2* promoter anchored-interaction to a sequence between ICR and CCD was detected on the paternal *ΔCCDIvr* allele (Fig 4A, boxed region; Sheet F in S5 Table). A stronger paternal-specific *Igf2* promoter looping interaction to this sequence may explain the observed strong increase in *Igf2* expression on the paternal versus the maternal *ΔCCDIvr* allele in adult skeletal muscle. Thus, we hypothesized that this sequence between ICR and CCD functions as a developmental- and tissue-specific enhancer to drive high *Igf2* expression on both the maternal and paternal *ΔCCDIvr* alleles.

### Evidence for a putative adult skeletal muscle enhancer (PaSME) between the ICR and CCD

To identify adult skeletal muscle enhancers at the *Igf2*/*H19* locus, we analyzed publicly available data characteristic of open chromatin, including DHS, assay for transposase-accessible chromatin with sequencing (ATAC) data, and histone enhancer marks (ChIP-seq H3K27ac and H3K4me2) across multiple tissues (Sheet A in S2 Table). Mouse skeletal muscle enhancer regions have been shown to be associated with coinciding H3K4me2 and H3K27Ac marks [38,39].

In neonatal/embryonic tissues, DHS marks the known SME in embryonic limb and neonatal heart, but not in neonatal liver (S4B Fig, green bar at SME). Similarly in embryonic limb, the putative enhancer region (~2 kb) captured by the *Igf2* promoter anchor on the paternal *ΔCCDIvr* allele in adult skeletal muscle (Fig 4A) demonstrated a modest limb-specific DHS peak that coincides with H3K27ac enrichment (S4B Fig). In adult skeletal muscle, this region exhibits stronger H3K27ac, H3K4me2, DHS and ATAC peaks, suggesting preferential enhancer activity in adults [38,40]. Furthermore, this sequence colocalizes with muscle-specific transcription factors and known markers of muscle-specific enhancers MYOD1 and MYOG during differentiation of C2C12 skeletal myoblasts to myotubes (Fig 4B and Sheet A in S2 Table). Taken together, these data suggest there is a ~ 2kb putative adult skeletal muscle enhancer sequence (designated PaSME) between the ICR and CCD (Fig 4B; S4B Fig, green bar at PaMSE). Collectively with Capture-C data, we hypothesize that 1) on the WT alleles, the CCD/Igf2Prox interaction blocks *Igf2* from accessing PaSME and 2) on both the maternal and paternal *ΔCCDIvr* alleles, PaSME is responsible for *Igf2* activation.

### Mouse 2D and 3D architecture at the TAD encompassing *Igf2*/*H19* locus is conserved in human cells

We next ascertained whether there was evidence for a PaSME-like region in human cells using publicly available human 3D and 2D chromatin data at the *IGF2*/*H19* locus. We retrieved 3D architecture data {chromatin interaction analysis by paired-end tag sequencing (ChIA-PET) [41]} visualized with Juicebox.js software (Sheet B in S2 Table), which depicts a CCD-CTCF dependent *IGF2* proximal-CTCF interaction (S5A Fig, dashed boxes; Sheet B in S2 Table) [42]. Secondary analysis further showed that CTCF dependent looping interactions from the *H19* ICR CTCF sites are maternal and interactions from the *IGF2* proximal CTCF sites are paternal [41]. CTCF site orientations and ChIP-seq binding are conserved at mouse *Igf2*/*H19* locus (S1 versus S5A Fig; Sheets D versus E in S2 Table). At the human ICR, one of CTCF sites originally defined [43] is not functional as defined by ChIP-seq binding (S5B Fig and Sheet E in S2 Table).

We also used publicly available DHS data across multiple adult tissues and ChIP-seq data for enhancer marks (H3K27ac, H3K4me2, H3K4me1) to identify muscle specific enhancers. Remarkably, adult muscle-specific enhancer marks and open chromatin at the murine PaSME are conserved at the orthologous human region in adult skeletal muscle (S5A Fig and Sheets B and C in S2 Table). Publicly available 2D data also indicate there is a putative adult skeletal muscle specific enhancer (PaSME) between the ICR and CCD at the human *IGF2*/*H19* locus (S5A Fig). Nevertheless, in both human and mouse the function of PaSME when CCD is intact, remains to be determined.

### Access to PaSME is not associated with high activation of nearby genes *H19*, *Nctc1* and *Mrpl23* in adult skeletal muscle

We hypothesized that (on the maternal allele) the ICR blocks the PaSME from engaging the *H19* promoter, analogous to how CCD blocks PaSME from engaging *Igf2* promoters. As expected, *H19* expression was unchanged on *ΔCCDIvr* alleles in neonatal and adult muscle (Fig 4C; S2D, S2E Fig). However, we anticipated *H19* would be highly elevated on *ΔICR* alleles in adult skeletal muscle, if unblocked PaSME engaged the *H19* promoter.

In neonatal muscle, *H19* and *Igf2* are expressed from the paternal and maternal *ΔICR* alleles at levels less than or equal to the WT alleles through sharing of the SME [5,6,26,27,31]. In adult GAS, *H19* and *Igf2* were modestly activated/elevated from the paternal and maternal *ΔICR* alleles, respectively (Fig 4D, 4E). (*H19* and *Igf2* levels from the respective maternal and paternal *ΔICR* alleles did not appear elevated.) These results suggest *H19* and *Igf2* on the *ΔICR* alleles are not under the control of an adult skeletal muscle specific enhancer activity, either from SME or PaSME. The outcomes indicate the PaSME (1) engages specific promoters (*Igf2* but not *H19*), or (2) activity is unidirectional, only engaging telomeric promoters.

We anticipated that genes immediately proximal to *H19* (non-coding *Nctc1* and *Mrpl23*) may be more accessible to the WT paternal PaSME compared to maternal PaSME due to the CTCF-bound maternal ICR functioning as an enhancer-blocker. Consistent with this expectation, PaSME anchored interactions to the *Igf2* promoter region were enriched on the adult paternal *ΔCCDIvr* allele (S4B Fig). Indeed, our data shows enrichment of interactions between paternal PaSME and the region surrounding *Nctc1* on the WT paternal allele compared to the maternal allele. If paternal PaSME were engaging with the *Nctc1* or *Mrpl23* promoters in a similar manner to the established enhancers for *Igf2/H19*, then we hypothesized that *Nctc1* and *Mrpl23* expression would be skewed towards the paternal allele. However, *Nctc1* and *Mrpl23* were biallelically expressed in adult skeletal muscle [31,44,45], suggesting that there are different regulatory elements contributing to *Nctc1* and *Mrpl23* expression.

## Discussion

**Fig 5:**
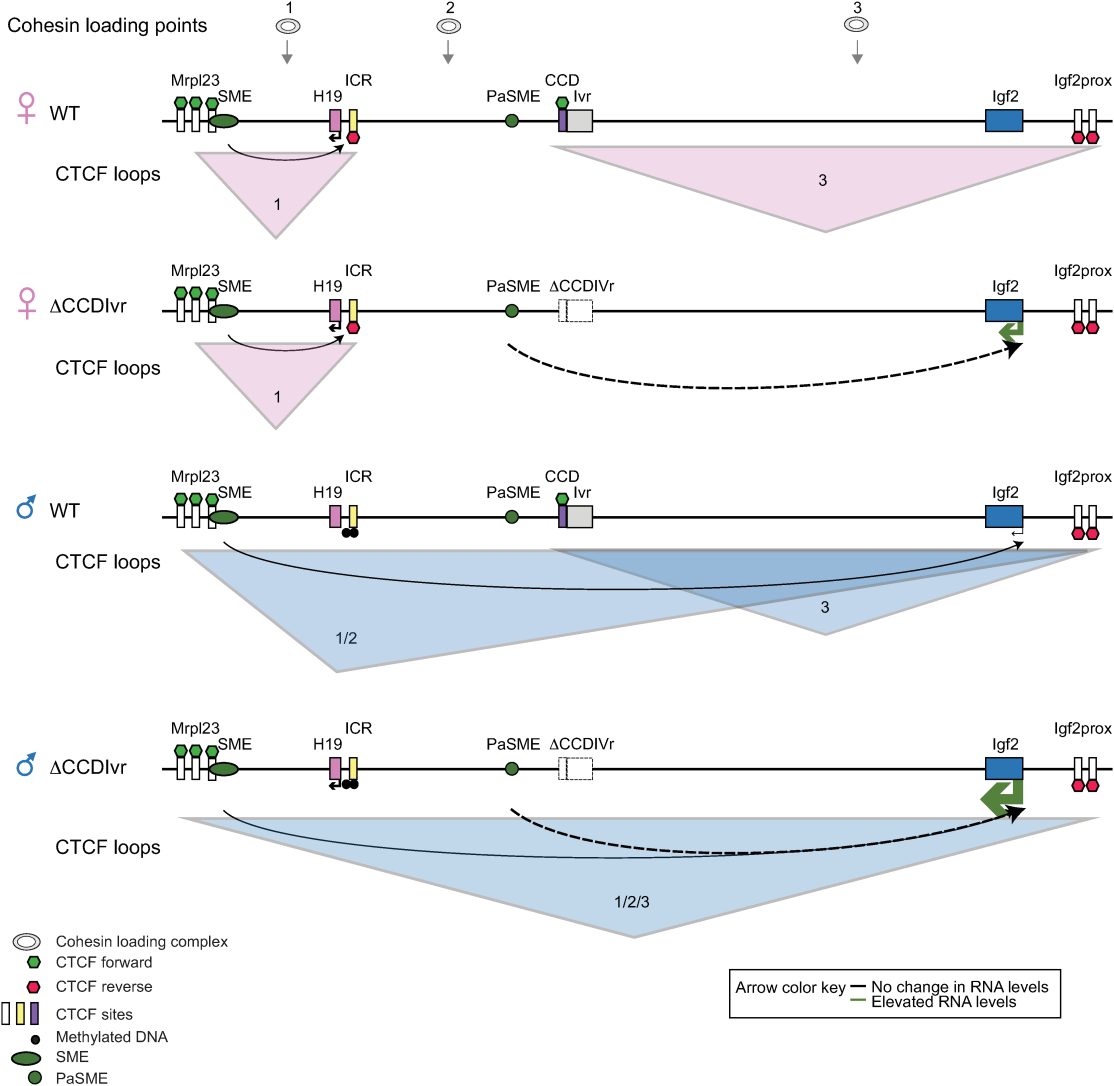
Model of *Igf2* accessing skeletal muscle enhancers (SME and PaSME) in adult SkM on maternal and paternal WT versus Δ*CCDIvr* alleles by cohesin loop extrusion and enhancer synergy. For cohesin complex loaded at regions 1, 2 and 3 (above alleles), cohesin loop extrusion model predicts formation of specific loops on the maternal (pink) and paternal (blue) alleles. Maternal ICR looping demarcates maternal *H19* exclusive access to the SME on both the WT and *ΔCCDIvr* maternal alleles, whereas CCD and Igf2prox looping insulates *Igf2* access to PaSKM on WT allele. We propose that an *Igf2* promoter gains access to PaSME (dashed line with arrow) on the maternal *ΔCCDIvr* allele. WT paternal Igf2prox forms loop with CCD (loop 3) and/ or with Mrpl23 CTCF sites (loop 1/2). SME most likely engages Igf2 SME within loop ½. On the paternal *ΔCCDIvr* allele, there is (1) increased probability of looping between Igf2prox and sequence near SME and (2) there is increased probability for an *Igf2* promoter to engage with SME and PaSME.

Here we uncover a mechanism by which CCD may repress *Igf2* by insulating *Igf2* from a PaSME. We first characterized the lncRNA, *Ivr*, adjacent to CCD, and determined that *Ivr’*s preferential paternal allele expression is likely due to sharing of *Igf2* enhancers that are centromeric to *H19*. Further, *Ivr* alone was not required to maintain CCD’s skeletal muscle *Igf2* repressor function. Deleting both the CCD and *Ivr* (*ΔCCDIvr* allele) partially activated maternal *Igf2* in neonatal skeletal muscle but generated extraordinary up-regulation of both maternal and paternal *Igf2* in adult skeletal muscle. Temporal and tissue specific 2D chromatin architecture data were used to identify a PaSME between ICR and CCD that is conserved at the syntenic human region. This 2D analysis, together with our allele-specific 3D architecture analysis of WT and Δ*CCDIvr* alleles, led us to propose that CCD dependent architecture blocks *Igf2* access to the PaSME located between the ICR and CCD. Below, we discuss properties of CCD (versus *Ivr*) and our model invoking cohesin loop extrusion and enhancer synergy to explain how *Igf2* accessibility to skeletal muscle enhancers SME and PaSME directs *Igf2* expression from WT and *ΔCCDIvr* alleles (Fig 5).

With the number of lncRNAs surpassing protein coding genes, efforts have focused on the role of lncRNAs [21]. Due to their low sequence conservation, elucidating lncRNA function is challenging [46]. As such, we did not find evidence for *IVR-*like transcription or gDNA conservation in the orthologous region adjacent to the conserved human CCD sequence. Whereas gene regulatory roles for several lncRNAs in *cis* and *trans* have been uncovered [21,46–50], *Ivr* was not required for normal allele-specific *Igf2* expression in neonatal and adult skeletal muscle (Fig 2A; S3B Fig, *Δ*Ivr** allele). For non-functional lncRNA transcripts, the regulatory region required for transcription, not the transcript, may dictate function [21,48,51]. Thus, we propose that cohesin and CTCF-bound *Ivr* promoter/regulatory region, CCD, may regulate *Igf2* expression in skeletal muscle as an architectural insulator (Fig 5 model). We cannot discount that the deleted *Ivr* sequence contributes to the *ΔCCDIvr* allele phenotypes, although deleting CCD sequence alone activated maternal *Igf2* in an early transgenic model [17].

Loop extrusion provides an explanation for the formation of cohesin guided chromatin CTCF loops, whereby cohesin complexes are loaded at potentially random sequences, and then loops of DNA are extruded until they are connected/stalled at convergent CTCF sites/boundary elements [35,52,53]. At the *Igf2*/*H19* locus, in neonatal and adult skeletal muscle, WT maternal and paternal-specific loops anchored from the ICR and Igf2prox CTCF sites (S1 Fig) were as predicted (Fig 5; maternal WT allele CTCF loops 1 and 3, paternal WT allele CTCF loops 1/2 and 3). On the maternal WT alleles, CTCF bound ICR forms a strong block to cohesin migration as loop interactions with Igf2prox anchor appear limited to CCD on the maternal WT allele (S1 Fig) and absent on the maternal *ΔCCDIvr* allele (Fig 3E; S3C Fig).

On the paternal allele, CTCF does not bind the hypermethylated ICR [54] and Igf2prox/*Mrpl23* loops are formed (Fig 5 -paternal allele 1/2 loop, S1 Fig).On both parental alleles, cohesin loaded at sequence between CCD and *Igf2* is predicted to direct the CCD/Igf2prox looping (Fig 5 [Loop 3]; S1 Fig). This Igf2prox/CCD CTCF loop insulates *Igf2* within the loop from enhancers outside of the loop. Because there are multiple CTCF sites at Igf2prox, the Igf2prox/CCD loop could form within the Igf2prox/Mrpl23 loop (Fig 5, WT paternal alleles). Our Capture-C analysis cannot determine if these two Igf2prox anchored loops were formed simultaneously in one cell or individually in different cells.

It is agreed that (1) E-P interactions require physical proximity and (2) enhancers and target genes should be within the same CTCF loop domain [55,56]. The SME engages maternal *H19* within the ICR anchored loops and paternal *Igf2* within Igf2prox anchored loops (Fig 5; S1 Fig). In contrast, the hypothesized PaSME, external to the Igf2prox/CCD loop, is blocked from engaging *Igf2* on both parental alleles. We propose that activation of *Igf2* on the maternal *ΔCCDIvr* allele in adult skeletal muscle is due to removal of the block to adult skeletal muscle enhancer activity (PaSME) (Fig 5). On the maternal *ΔCCDIvr* allele (Fig 3E; S3C Fig), our data suggest that no new Igf2prox anchored loops are formed that would facilitate PaSME engaging *Igf2* promoters. On the paternal *ΔCCDIvr* (versus WT) allele in adult skeletal muscle, Igf2prox/Mrpl23 interactions and *Igf2* promoter contacts to both SME and PaSME are strengthened (Fig 3E, 4A, 5). We propose that high *Igf2* activity on the paternal versus maternal *ΔCCDIvr* allele (Fig 3D versus 3B) is due to SME and PaSME acting in synergy to activate adult *Igf2* (Fig 5) [55,57].

Because CTCF loops are dynamic and some E-P interactions are not cohesin-dependent, loop extrusion does not fully explain E-P interactions [52,58,59]. (1) How might CCD block PaSME activity independent of Igf2prox/CCD loop formation on the paternal WT allele (Fig 5, Loop 1/2)? (2) How does PaSME access *Igf2* promoters on the *ΔCCDIvr* allele? The *Igf2* promoter/PaSME interaction could be mediated by CTCF/cohesin-independent mechanisms that bridge E-P interactions by use of self-interacting DNA associated proteins and transcription factor hubs at enhancer regions [60]. Furthermore, deconstructing the regulatory controls of enhancer-bound and promoter-bound proteins that cause these elements to interact may uncover why access to PaSME results in high levels of *Igf2* activation on the *ΔCCDIvr* alleles (Fig 3B, 3C, 3D), but minimal change in *H19* expression on the *ΔICR* alleles (Fig 4C) [56]. Alternatively, PaSME activity may be orientation-dependent, as has been demonstrated for an enhancer at globin locus [61], and therefore unblocked PaSME engages with telomeric *Igf2* but not centromeric *H19*. We anticipate advancement of high-resolution live cell imaging technologies that simultaneously report between 3-D architecture and gene transcription in cells of interest are required to decipher these predicted outcomes [59,62]. Deletion of PaSME in the presence and absence of CCD*Ivr* will validate the role of PaSME in adult skeletal muscle.

The *ΔCCDIvr* mice are a model for how tissue-specific overexpression of *Igf2* can affect skeletal muscle development in adults, when *Igf2* is normally repressed. *In vitro* studies have shown that *Igf2* plays a role in myoblast differentiation, however, our understanding of *Igf2* in muscle development and regeneration *in vivo* is limited [63,64]. Global prenatal transgene *Igf2* overexpression led to perinatal lethality, whole body overgrowth and other Beckwith-Wiedemann syndrome phenotypic determinants [65]. Transgenic models with persistent adult tissue-specific *Igf2* overexpression led to localized overgrowth and/or cancer, uncovering autocrine and endocrine mechanisms of action [66,67]. In a Duchenne Muscular dystrophy mouse model, muscle degenerative phenotypes associated with lack of dystrophin were ameliorated by elevated serum IGF2 peptide from one of these transgenes [67,68]. While some studies have suggested that approaches to elevate muscle IGF2 peptide availability could be therapeutic for the treatment of muscular dystrophies [64,68], one indicated it would be detrimental [9]. Defining metabolic and physiological outcomes and IGF2 autocrine signaling consequences of *Igf2* overexpression in *ΔCCDIvr* adult skeletal muscle could resolve these possibilities.

In summary, we demonstrate that CCD*Ivr* sequence functions as a skeletal muscle repressor by forming a loop with *Igf2* proximal CTCF sites and thereby isolating *Igf2* within the loop from skeletal muscle enhancers outside of the loop. The *Igf2*/*H19* locus continues to be an excellent model for deciphering how allele-specific genome architecture facilitates enhancer-promoter interaction that dictate tissue-specific gene expression.

Limitation of Capture C analysis: (1) Few deletion allele samples were analyzed, therefore we could not evaluate statistical significance for the neonatal maternal and paternal *ΔCDDIvr* alleles and the adult maternal *ΔCDDIvr* allele. (2) From the capture-C libraries we achieved a read length up to 300nt (we had anticipated reads up to 500nt). The achieved read lengths were not long enough to capture connections with *Mrpl23* and SME CTCF site anchored probes (Sheets A and E in S4 Table) Nevertheless, from the Igf2prox, *Igf2* promoter, CCD and PaSME anchored probes we observed similar changes interactions peaks on individual *ΔCDDIvr* alleles (compared to the WT alleles) that were measured for the adult paternal *ΔCDDIvr* (2 samples, S5 Table).

## Materials and methods

### Ethics statement

Mouse studies were approved by and performed in accordance with the Institutional Animal Care and Use Committee (IACUC) at the University of Pennsylvania (Protocol Number: 804211).

### Animal

Briefly, male and female C57BL/6J (JAX:00664) (B), E2a-Cre (JAX:003724) and ROSA26::FLPe (JAX:016226) mice were obtained from Jackson Laboratories to generate new alleles (*ΔIvr + Neo*, *ΔIvr* and *ΔCCDIvr*). Cast7 (C) mice were also sourced from Jackson Laboratory and used to generate progeny for allele-specific analysis [69]. Mouse model Δ3.8kb-5’H19 (here designated *ΔICR*) [26] was used for comparative analyses with our newly derived *ΔCCDIvr* line. All mice with the deletion alleles (mouse lines *ΔIvr + Neo*, *ΔIvr* and *ΔCCDIvr*) were maintained on a B background and characterized as described below. These mice were backcrossed into C57BL/6J for at least 6 generations. When not breeding, up to five adult mice were housed in individually ventilated cages with a 12-hour light/dark cycle at a temperature of 22°C and relative humidity of 50%. All mice were maintained in a pathogen-free facility and housed in polysulfone cages. Mice had *ad libitum* access to standard laboratory chow (Laboratory Autoclavable Rodent Diet 2010, LabDiet), drinking water, and environmental enrichment (i.e., nesting material was provided to enhance their welfare).

Beginning at weaning, body weight measurements of adult mice were recorded, on average, every 2 weeks. Prior to tissue collection, neonates were euthanized by decapitation and adults were euthanized by cervical dislocation. For initial survey analysis, skeletal muscle subtypes including gastrocnemius, quadriceps, tibialis anterior and tongue were collected from 7 weeks-old male mice (Fig 2D). For subsequent analyses, the skeletal muscle subtypes extensor digitorum, gastrocnemius, quadriceps, soleus, tibialis anterior and tongue were collected from 4-5 month-old adult mice. For expression analyses, a minimum of 3 mice of each genotype (WT and maternal- or paternal-specific inheritance of deletion alleles) were used. Adult male mice tissues were processed for expression analyses. All tissues were snap-frozen when collected, unless used for nuclei isolation as described below. Frozen tissues were stored at -80°C.

### Target vector

The target vector was designed to delete the *Ivr* and introduce *loxP* sites for Cre-mediated deletion of CCD. The target vector arms (5’ arm - included CCD sequence [*mm10 chr7*: 142,604,115–142,611,247]; 3’ arm – included sequence adjacent to *Ivr* coding sequence [*mm10 chr7*:142,620,880–142,624,437]) were amplified from 129SvEvBAC genomic DNA (bMQ318o12) by iProof High Fidelity DNA Polymerase (BIO-RAD, Hercules CA USA) and cloned into BluescriptIIKS. Following published guidelines [70–72], recombineering was used to introduce (1) a *loxP* site on the target vector 5’ arm (replaced G at *mm10 Chr7*: 142608373) and (2) a *Frt-*flanked *neo* cassette plus a *loxP* site between the 5’ and 3’ target vector arms. Finally, for negative selection, a 2.3kb *Sal*I diphtheria toxin A cassette (DTA) [73] was cloned adjacent to the 3’ target vector arm. The vector was linearized by *Not*I digestion. See S2 Fig for the target vector schematic.

### TALEN plasmids

We assembled transcription activation-like effector nuclease (TALEN) plasmids to facilitate targeting at the *Ivr* locus. We used Paired Target Finder to design TALENs to minimize off-target recognition [74] (https://tale-nt.cac.cornell.edu/). TALENs were designed to target double-stranded breaks of endogenous sequence that were not present in the target vector (i.e., 5’ target vector arm where *loxP* site is inserted and *Ivr* sequence that is designed to be deleted by the targeting event). We assembled TALENs using the TALE Toolbox system [75]. (See S2 Fig for location of TALEN targets: on 5’arm TALENs were generated for targeting ‘caccccctttccttgctga’ and ‘tttctttcatggccccta’; on 3’ arm TALENs were generated for targeting ‘caccccctttccttgctga’ and ‘tttctttcatggccccta’.)

### Generation of *ΔIvr + neo* allele in ESCs

Linearized target vector was electroporated into E14.1 ESCs [76] to generate Δ*Ivr* + *neo* ESCs using previously described cell culture, electroporation and Southern blot analysis methods [77], with the following exceptions. To facilitate targeting, TALEN plasmids targeting CCD*Ivr* locus were electroporated with the target vector (S2 Fig). Genomic DNA (gDNA) isolated from G418-resistant positive clones was digested with *Dra*I and analyzed by Southern blots with 5’ and 3’ external probes (S2 Fig and Sheet A in S3 Table).

### Generation of mice with the *ΔIvr + Neo*, *ΔIvr* and *ΔCCDIvr* alleles

ESC clones heterozygous for the *Δ*Ivr + Neo** allele were provided to the Transgenic and Chimeric Mouse Facility in the Department of Genetics at the University of Pennsylvania for injection into blastocysts to derive chimeric mice. Chimeric mice were bred to C57BL/6J and agouti progeny inheriting the *Δ*Ivr + Neo** allele were bred to ROSA26::FLPe knock-in mice (RRID:IMSR_JAX:016226) to remove the *Frt*-flanked neomycin-resistance cassette (neo) to generate progeny with the *Δ*Ivr** allele. Mice with the *Δ*Ivr + Neo** allele or *Δ*Ivr** allele were bred to *E2a-Cre* transgenic mice (RRID:IMSR_JAX:003724) to remove *loxP*-flanked CCD sequence to generated progeny with the *ΔCCDIvr* allele.

### DNA isolation and genotyping

DNA was extracted from ESCs [77], mouse ear punches [78] and mouse tissues [79]. Primers and conditions used for PCR genotyping *E2a-Cre*, *ROSA26::FLPe*, *ΔICR*, *Δ*Ivr + Neo**, *Δ*Ivr** and *ΔCCDIvr* alleles are provided in Sheet B in S3 Table [26,80]. The maternal allele is listed first and the paternal allele is listed second for all genotypes.

### RNA isolation

RNA was extracted from frozen tissues in TRIzol (15596026, Thermo Fisher Scientific, Waltham, MA, USA) using manufacturer’s instructions. This included homogenization of 50–100 mg tissue in 1 ml TRIzol with a Polytron homogenizer (Binkmann Polytron Kinematica PT 10–35) for 25–30 seconds, optional centrifugation of homogenate to collect cleared supernatant and optional addition of 1 μl GlycoBlue (AM9516 Invitrogen) to the aqueous phase following chloroform extraction. RNA was then precipitated and purified from the aqueous phase on a RNAeasy Mini kit column following manufacturer’s instructions (Qiagen, Hilden, Germany). gDNA was removed from RNA using RQ1 RNase-Free DNase (Promega, Madison, WI, USA) using manufacturer’s instructions.

### *Ivr* 5’RACE-PCR

In the laboratory of Dr. Denise Barlow [22], 5’RACE was performed with FirstChoice RLM-RACE Kit, with two rounds of nested PCR for 5’RACE. 5’RACE-PCR products were gel extracted and then cloned into pGEM-T for sequence analysis. See Sheet A in S1 Table for 5’RACE primers and pGEM-T 5’RACE clone sequences.

### cDNA synthesis, allele-specific expression and total RNA

cDNA synthesis, reverse transcription quantitative polymerase chain reaction RT-qPCR and allele-specific expression analyses were performed as previously described [81]. Allele-specific expression primers with RFLP outcomes are provided in Sheet C in S3 Table [81,82]. Total RNA levels for all genes were quantified by RT-qPCR and measured relative to the geometric mean of RNA levels of *Arbp* (*acidic ribosomal phosphoprotein P0*), *Nono* (*non-POU domain-containing, octamer-binding protein*) and *Rpl13a* (*ribosomal protein L13A*). For RNA levels reported on y-axis, the mean value of wild-type measurements was set as 1. Primers for RT-qPCR are provided in Sheet D in S3 Table [81].

### Statistical analysis or analyses

Differences between two groups were evaluated by unpaired t test with Welch’s correction using GraphPad Prism software: *p < 0.05, **p < 0.01, ***p < 0.001, ****p < 0.0001. For Capture C data, statistical analyses are described below.

### CTCF binding site and orientation analysis

The most likely CTCF binding sites within DNA fragments (~150–300 bp) within predominant CTCF ChIP-seq peaks (across mouse region mm10 Chr7:142,300-000-142,750,000 and at human region hg19 Chr11:1,645,000–2,225,000) were analyzed. The most likely CTCF binding and CTCF motif orientation was determined using the PWMScan – Genome Position Weight Matrix (PWM) scanner (https://epd.expasy.org/pwmtools/pwmtools/pwmscan.php) [83] and CTCF consensus sequence from the JASPAR CORE 2020 motif library [84] to extract the matching score for the best motif instance at each binding site. Human CTCF motif orientation was also determined with the PWMScan. See Sheets D and E in S2 Table for method details.

### Nuclei isolation for capture C

Nuclei were isolated from fresh neonatal and adult mouse hindlimb muscle from hybrid WT mice (BxC and CxB) and *ΔCCDIvr* mice (*ΔCCDIvr* x C and C x *ΔCCDIvr*) (Sheet B in S4 Table). Neonatal hindlimb nuclei were isolated as described by Juan et al. 2022 with a few modifications [85]: Isolated hindlimb muscle was placed on ice in a 1.5ml Eppendorf tube, covered with ice-cold homogenization buffer (10 mM Tris-HCl, pH 8.0; 5 mM CaCl_2_; 3 mM MgAc_2_; 0.32 M sucrose; 0.1% Triton-X; 0.1 mM EDTA, 1X Protease Inhibitor cocktail without EDTA (Roche); 1mM DTT; 0.1 mM PMSF) and then minced using a fine scissor. Minced muscle was transferred to 10 ml homogenization buffer in a 15 ml tube and dounced 21 times with loose-fitting pestle and then 14 times with tight -fitting pestle. Released nuclei were cleared of debris by filtration into 50 ml conical tubes through a series of pre-rinsed cell strainers (100 μm, 70 μm, 40 μm and 20 μm) as adapted from Nohara et al. 2017 [86]. At room temperature, the cleared nuclei were then fixed on a rotating platform in 1% formaldehyde for 10 minutes and fixation was stopped with glycine at a final concentration of 0.125 M on rotating platform for 5–10 minutes. Nuclei were pelleted at 500 x g for 15 minutes at 4°C. Then nuclei were washed with 50 ml ice-cold PBS and again centrifuged at 500 x g for 15 minutes at 4°C. Nuclei were resuspended in ~2 ml cold PBS, counted, transferred to a LOW BIND Eppendorf (≥ 5 ml) tube and centrifuged at 700 x g for 10 minutes at 4°C. Pellet was snap frozen in liquid nitrogen and stored at -80°C.

All steps to isolate adult hindlimb nuclei were performed in a cold room with reagents stored and procedures performed on ice, except for fixing of nuclei. Gastrocnemius, tibialis anterior and quadriceps muscle were dissected, placed in 2ml tube, covered with freshly prepared hypotonic buffer (10 mM HEPES-KOH (pH 7.3, 10 mM KCl, 5 mM MgCl_2_, 0.1% NP-40, 0.1 mM PMSF, Protease Inhibitor cocktail (PIC) 1X, 1 mM DTT) and then minced with fine scissors. The methods and hypotonic buffer (with addition of 1 mM DTT) of Joshi et al. 2017 [87] were used, with slight modifications. Minced muscle was added to a 50 conical tube with 10 ml hypotonic buffer and homogenized with a mechanical tissue homogenizer for ~10 seconds. Hypotonic buffer was added to dilute homogenate to 30 ml, homogenate was added to 40 ml dounce tube and incubated for 5 minutes on ice and then dounced with loose pestle 10 times. Lysate was divided into two 50 ml conical tubes, transferred to room temperature, and fixed as described above for the neonatal nuclei isolation. The tubes of fixed lysate were recombined and then returned to the cold room where it was further dounced 10 times with the loose pestle, pelleted at 1,000 x g for 5 minutes at 4°C and then resuspended in 10 ml of hypotonic buffer. Lysate was then filtered through cell strainers as described above for neonatal nuclei, adapting adult muscle nuclei filtration protocols [86,87]. Nuclei were counted and filtrate was transferred to a 15 ml LOW BIND Eppendorf tube, centrifuged at 1,000 x g for 5 minutes at 4°C. Pellet was snap frozen in liquid nitrogen and stored at -80°C.

**Next generation Capture-C sequencing and statistical analysis Capture-C** The Capture-C was as described [85]. Nuclei were isolated from neonatal and adult hindlimb muscle obtained from reciprocal hybrid WT mice (BxC and CxB) and heterozygous mutant *ΔCCDIvr* mice. We constructed, fragmented and indexed 3C library, performed oligonucleotide capture on pooled libraries, and performed sequencing and analyses of captured sequences, as previously described [85], with the following modifications. Here, the biotinylated oligonucleotides were designed for multiple CTCF binding sites across the *Igf2*/*H19* locus (including the ICR), *Igf2* promoters and previously identified and putative enhancer skeletal muscle enhancers using IDT Discovery Pool technology (Sheets A and C in S4 Table). The first hybridization reaction included five pools described by Juan et al. and two additional pools described herewith (one pool assayed the four neonatal muscle samples; the other pool assayed the six adult muscle samples [see Sheets D and E in S4 Table]), which were each processed as previously described [85].

Bioinformatic analysis of the resulting reads was conducted as previously described [85], with the following modifications to implementing the CCseqBasicS pipeline. The CCseqBasic tool (vCC5) tool was used in both non-SNP-specific as well as SNP-specific configurations, however, here C57BL/6J (B)/Castaneous (C) SNPs were evaluated. (Most designed probes had B/C SNPs nearby to be sequenced; the list of used SNPs is available in Sheet C in S4 Table). The total read counts for each probe region, including non-SNP-specific regions and regions for each SNP (C, B), are provided in Sheet D in S4 Table. Bigwig files were generated for each sample and each probe by piling up all reads, B reads and C reads separately, and then normalized to 10K reporter reads per designed probe for track visualization. We pooled reads from alleles in the same experimental group (WT.neonatal.maternal, WT.neonatal.paternal, WT.adult.maternal, WT.adult.paternal, [CCDIvr pKO] KO.adult.paternal) together for calling contact sites, statistical analysis and visualization (Sheets K and L in S5 Table). For each experimental group and for each probe, we defined contact sites in two ways: the “given coordinates” approach which we manually defined contact sites (loci with CTCF motif, *Igf2* promoters, previously reported skeletal muscle enhancer and a putative skeletal muscle enhancer [Sheets A-J in S5 Table]), and the “peak calling” approach for which we used computational algorithm to call contact sites from the data. More specifically for the peak calling approach, contact sites were defined as peaks called using MACS2 (v2.1.1) with parameters “--nomodel --extsize 1200 --qvalue 1e-100 --broad --nolambda --broad-cutoff 1e-100 --minsize 5000”. The corresponding contact site visualization, quantification of contact sites, differential analysis between experimental groups and generation of figures on WashU Epigenome browser was performed as previously described [85]. Statistics for locus-specific comparisons are provided in S5 Table for both the “peak calling” and “given coordinates” approach.

### Data and code availability

The accession number for raw and processed Capture-C sequencing data reported in this paper is publicly available on date of publication and is deposited at GEO:GSE202669. We examined existing, publicly available data provided by the ENCODE Project using the New WashU Epigenome Browser, visualized on the *mouse mm10* and *human hg19* genomes (Sheets A and B in S2 Table). Additional supporting data is provided in S1 and S2 Data files. We do not report original code.

## Supporting information

S1 Fig CCD association with *Igf2* within the *Igf2*/*H19* locus TAD (UCSC browser mm10 chr7:142,350,000–142,700,000). From top to bottom, the following are depicted: Genes, regulatory elements (SME, EE, ICR and CCD) and probes are annotated (Sheet C in S2 Table). *Ivr* overlaps with predicted (GM33148) lncRNA. CTCF binding site (BS) polarity (green forward arrow/bars for forward, and red reverse arrow/bars for reverse) is designated with WashU Epigenome Browser tracks using Public ENCODE and 4D Nucleome Network data (Sheet A in S2 Table). Micro-C interactions between CCD and CTCF sites proximal to *Igf2* (Igf2prox) are highlighted (dashed lines). Purple arrow heads mark Igf2prox interaction that are detected by the following Capture-C analysis. Below are normalized Capture-C interaction frequencies of WT F1 hybrid neonatal and adult skeletal muscle from the viewpoints probes (anchors) at CCD, at Igf2prox, and at the ICR (Sheets A-D in S5 Table). Interactions between CCD (biallelic) and Igf2prox (maternal and paternal) are highlighted (purple vertical bars). Black tracks designate interactions from anchor (CCD) without an available polymorphism to discriminate alleles. Pink and blue tracks designate maternal and paternal alleles, respectively, and darker intensities correspond to stronger interactions. Selected allele-specific interactions are boxed (paternal Igf2prox to the SME region, maternal ICR to the SME region, maternal proxKrtap5–4 to ICR and paternal proxKrtap5–4 to proxIgf2) with Wald statistics using two methods (* or # p < 0.05, ** or ## p < 0.01, *** or ### p < .001, #### p < .0001, Sheets A-D in S5 Table). Probe and regulatory regions are also annotated below the Capture C data.
(PDF)

S2 Fig Generation of Δ*Ivr* and Δ*CCDIvr* alleles and neonatal analysis. (A) UCSC genome browser view of *Igf2*/*H19* locus with mapped CCD, conserved regions CR1 and CR2, DHS, *Ivr*Ex1, *Ivr*Ex2 and *Ivr*Ex3 sequence (Sheet C in S2 Table), above, and targeting vector and targeted loci *ΔIvr + neo*, *ΔIvr* (after Flp mediated removal of neo) and *ΔCCDIvr* (after Cre mediated removal of CCD), below. *Rr1*^*em1.1Msb*^, *Rr1*^*em1.2Msb*^ and *Rr1*^*em1.2Msb*^ are the MGI approved nomenclature for the respective targeted alleles. The endogenous locus (thick black line) depicts endogenous *Dra*I sites, the sites where *Dra*I and loxP sequences (pink bars) were introduced (insert site), TALEN cut sites (T), CCD (purple box), *Ivr* transcription unit (gray boxes), targeting vector 5’ and 3’arms (open boxes) and the 5’ and 3’ sequence used for Southern blot analyses (5’P and 3’P, dark gray boxes). In addition to the 5’ and 3’ arm (thick black line), the targeting vector includes introduced *Dra*I site, loxP sites (pink bars), Frt (blue bars) flanked Neo-cassette (open box), DT cassette (open box), and BSSKII (dashed line). Primer pairs (c, d, e, f) used to genotype the targeted loci before and after neo and CCD removal are depicted below the endogenous and target loci. (B) *Dra*I digested DNA analyzed by Southern blots. Using the 5’P probe, the 18.2kb endogenous and 8.9kb targeted *Dra*I fragments are detected. Using the 3’P probe, the 7.2 endogenous and 8.9kb, 7.1kb and 4.1kb target *Dra*I fragments are detected. (C) Total *Igf2* expression in *CCDIvr*^*+/+*^ versus *CCDIvr*^*+/Δ*^ (paternal *ΔCCDIvr*) neonatal tongue. (D) Allele-specific *H19* expression in neonatal liver (Li), tongue (T) and heart (H) from WT and *ΔCCDIvr* alleles. *H19* qRT-PCR products are digested with *Cac8*I to discriminate the parental B (WT B, *ΔCCDIvr*) and C alleles. (E) Total *H19* expression in *CCDIvr*^*+/+*^ versus *CCDIvr*^*Δ/+*^ (maternal *ΔCCDIvr*) neonatal tongue. (F) Weights of WT (*CCDIvr*^*+/+*^) versus maternal deletion (*CCDIvr*^*Δ/+*^) and WT (*CCDIvr*^*+/*+^) and paternal deletion (*CCDIvr*^*+/Δ*^) neonatal littermates. No statistical difference between weights of mutant and WT littermates.
(PDF)

S3 Fig Analyses of adult and neonatal mice. (A) For adult, weights of WT versus mice inheriting the maternal *ΔCCDIvr* allele (top panels) and WT versus mice inheriting the paternal *ΔCCDIvr* allele (bottom panels) (Two-tailed Welch’s t-test: *p < 0.05, **p < 0.01, ***p < 0.001; error bars represent SD). (B) Allelic *Igf2* expression in adult TA from mice inheriting the *ΔIvr* allele. *Igf2* RT-PCR products were digested with *MluC*I to discriminate the parental B (WT B, *ΔIvr*, *ΔCCDIvr*) and C alleles. (C) and (D) All data is presented on WashU Epigenome Browser mouse mm10: chr7:142,300,000–142,750,000. (C) From top to bottom, the following are depicted: Genes, regulatory elements (SME, ICR, CCD) and probes are annotated (Sheet C in S2 Table). WashU Epigenome Browser CTCF ChIP Seq tracks (Sheet D in S2 Table). Below are normalized Capture-C interaction frequencies of F1 hybrid neonatal SkM from the viewpoint of anchors at CCD and at Igf2prox (Sheet F in S5 Table). CCD and Igf2prox anchored regions are highlighted (purple bars). Black tracks designate interactions from anchor (CCD) without an available polymorphism to discriminate alleles. Pink and blue tracks designate maternal and paternal alleles, respectively, and darker intensities correspond to stronger interactions. The biallelic interaction between sequence adjacent to CCD and Igf2prox occurs on the wild-type allele but is absent from *ΔCCDIvr* alleles in neonatal SkM. Normalized Capture C tracks to/from Igf2prox and CCD anchors (purple bars) on wild-type (WT, WT_maternal or WT_paternal) and *ΔCCDIvr* (MKO_maternal and PKO_paternal) alleles in neonatal SkM. Probe and regulatory regions are also annotated below the Capture C data.
(PDF)

S4 Fig *Igf2* promoter interactions, PaSME interactions and enhancer marks in neonatal (and adult) skeletal muscle. (A) and (B) All data is presented on WashU Epigenome Browser mouse mm10: chr7:142,300,000–142,750,000. Presented from top: annotated genes, regulatory elements and probes and WashU Epigenome Browser CTCF ChIP Seq track (Sheet A and C in S2 Table and Sheet A in S4 Table). Normalized allele-specific Capture-C peaks on WT, MKO and PKO alleles. (A) Captured interactions from anchor at *Igf2* promoter. As measured on WT alleles, boxed regions depict paternal specific interaction of *Igf2* promoter to *Nctc1*/SME region (green bar) (Sheet G in Table 5). (B) Analysis of putative adult skeletal muscle specific enhancer (PaSME) region (green bar) in embryonic and neonatal limb (Sheet A in S2 Table, mm10 chr7:142599494–142601493). All data presented on WashU Epigenome Browser mouse mm10 and as referenced in Sheet A in S2 Table. ENCODE DNase-seq (DNase Hypersensitivity Site, DHS) data from neonatal heart and liver and embryonic limb, and H3K27ac and H3K4me2 ChIP-seq peaks in embryonic limb are shown. Below, captured interactions from anchor at PaSME in neonatal and adult skeletal muscle. On WT alleles, boxed regions depict paternal specific interaction to *Nctc1*/SME region (‘a’ significant, ‘b’ trending, Sheets H and I in S5 Table). On adult PKO allele, a boxed region at *Igf2* promoter indicates significant enhancer-promoter (E-P) interactions (Sheet J in S5 Table). SME and PaSME regions are highlighted with green bars and *Igf2* promoter region is highlighted with a blue bar. (A) and (B) Wald statistics using two methods to identify peaks are presented (* or # p < 0.05, ** or ## p < 0.01, Sheets G, H and J in S5 Table).
(PDF)

S5 Fig Conservation of CTCF binding and chromatin structure at human *IGF2*/*H19* locus. (A) 3D and 2D chromatin structure at human *IGF2*/*H19* locus (hg19 chr11:1,645,000–2,225,000). Source of publicly available data is presented in Sheet B in S2 Table. Juicebox software 3D architecture data [chromatin interaction analysis by paired-end tag sequencing (ChIA-PET)] in human GM12878 cells. The conserved interaction between CCD and proximal *Igf2* CTCF sites is presented in dashed boxes. RefSeq Genes, CCD, CTCF ChIP-seq peaks (GM12878 cells, ENCODE) are noted to left and on top of Juicebox interaction map. Below are UCSC genome and WashU browser views of genes within hg19 chr11:1645000–2225000. Location of ICR, CCD, conserved regions hCR1 and hCR2 (upper panel) are designated (Sheet C in S2 Table). CTCF site polarity (green forward arrow/bars for forward, and red reverse arrow/bars for reverse) and CTCF and RAD21 ChIP seq data for indicated cell lines are presented in upper panel (Sheets B and E in S2 Table). In lower UCSC genome browser view, with annotated mouse homologous SME and PaSME regions (green bars), DNase-Seq (DHS) and ChIP-Seq (H3K27ac, H3K4me2, H3K4me1) tracks from designated adult tissues are presented. The Juicebox and UCSC and WashU browser genomes are aligned with each other. (B) CTCF is not bound to originally annotated CTCF binding site (BS) CTS5 in human cell lines; CTCF binding is shown at a newly annotated CTCF BS ICR7 (Sheet E in S2 Table).
(PDF)

S1 Table Sheet A_*Ivr* 5’RACE-PCR clones; Sheet B_*Ivr* Ex2_Ex3 Splice Junction. (XLSX)

S2 Table Sheet A_Mouse Public Data; Sheet B_Human Public Data; Sheet C_Genome Coordinates for ROI; Sheet D_PWM Mouse CTCF ChIPSeq Motifs; Sheet E_PWM Human CTCF ChIPSeq_Motifs. (XLSX)

S3 Table Sheet A_Southern blot probes; Sheet B_Genotyping primers and pcr conditions; Sheet C_Allele-specific expression primers and RFLP assays; Sheet D_Total RNA (RT-qPCR) primers. (XLSX)

S4 Table Sheet A_Capture-C Probes (oligonucleotides); Sheet B_Nuclei processed for CapC; Sheet C_CapC SNPs; Sheet D_Preprocessing Stat; Sheet E_Reporter Reads. (XLSX)

S5 Table Sheets A-J_Statistics for designated figure sample comparison and CapC anchor; Sheet K_Neonatal Comparison Sample Summary; Sheet L_Adult Comparison Sample Summary. (XLSX)

S1 Data Raw Images. (PDF)

S2 Data Graph data and statistics. (XLSX)
