# Supplementary material for: Igf2 adult-specific skeletal muscle enhancer activity revealed in mice with intergenic CTCF boundary deletion
Source: PLoS Genet. 2025 Aug 29;21(8):e1011834. doi: 10.1371/journal.pgen.1011834 (PMC12416839; doi:10.1371/journal.pgen.1011834)
Supplement: S1 Fig — From top to bottom, the following are depicted: Genes, regulatory elements (SME, EE, ICR and CCD) and probes are annotated (Sheet C in S2 Table). Ivr overlaps with predicted (GM33148) lncRNA. CTCF binding site (BS) polarity (green forward arrow/bars for forward, and red reverse arrow/bars for reverse) is designated with WashU Epigenome Browser tracks using Public ENCODE and 4D Nucleome Network data (Sheet A in S2 Table). Micro-C interactions between CCD and CTCF sites proximal to Igf2 (Igf2prox) are highlighted (dashed lines). Purple arrow heads mark Igf2prox interaction that are detected by the following Capture-C analysis. Below are normalized Capture-C interaction frequencies of WT F1 hybrid neonatal and adult skeletal muscle from the viewpoints probes (anchors) at CCD, at Igf2prox, and at the ICR (Sheets A-D in S5 Table). Interactions between CCD (biallelic) and Igf2prox (maternal and paternal) are highlighted (purple vertical bars). Black tracks designate interactions from anchor (CCD) without an available polymorphism to discriminate alleles. Pink and blue tracks designate maternal and paternal alleles, respectively, and darker intensities correspond to stronger interactions. Selected allele-specific interactions are boxed (paternal Igf2prox to the SME region, maternal ICR to the SME region, maternal proxKrtap5–4 to ICR and paternal proxKrtap5–4 to proxIgf2) with Wald statistics using two methods (* or # p < 0.05, ** or ## p < 0.01, *** or ### p < .001, #### p < .0001, Sheets A-D in S5 Table). Probe and regulatory regions are also annotated below the Capture C data. (PDF) [file pgen.1011834.s001.pdf]

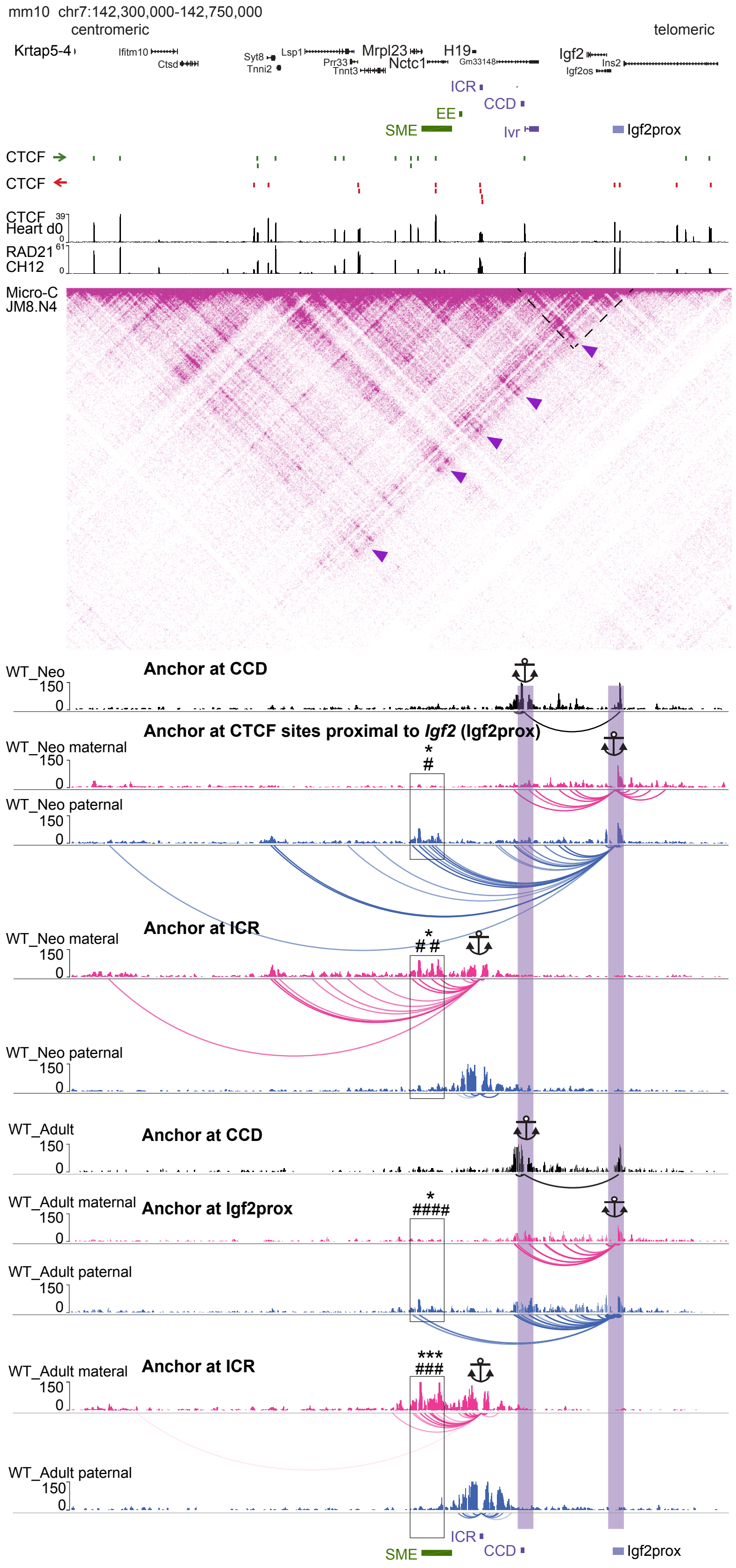

mm10 chr7:142,300,000-142,750,000
centromeric
telomeric
Krtap5-4
Ifitm10
Ctsd
Syt8
Tnni2
Lsp1
Prr33
Tnnt3
Mrpl23
Nctc1
H19
Gm33148
Igf2
Igf2os
Ins2
ICR
CCD
EE
SME
Ivr
Igf2prox
CTCF
CTCF
CTCF Heart d0
RAD21 CH12
Micro-C JM8.N4
WT_Neo
Anchor at CCD
Anchor at CTCF sites proximal to Igf2 (Igf2prox)
WT_Neo maternal
WT_Neo paternal
Anchor at ICR
WT_Neo materal
WT_Neo paternal
WT_Adult
Anchor at CCD
WT_Adult maternal
Anchor at Igf2prox
WT_Adult paternal
WT_Adult materal
Anchor at ICR
WT_Adult paternal
150
0
*
#
##
####
***
###
ICR
CCD
SME
Igf2prox
