# Supplementary figures and images for: Igf2 adult-specific skeletal muscle enhancer activity revealed in mice with intergenic CTCF boundary deletion
Source: PLoS Genet. 2025 Aug 29;21(8):e1011834. doi: 10.1371/journal.pgen.1011834 (PMC12416839; doi:10.1371/journal.pgen.1011834)

## A

### Adult mouse weights

#### Maternal $\Delta$ CCDIvr

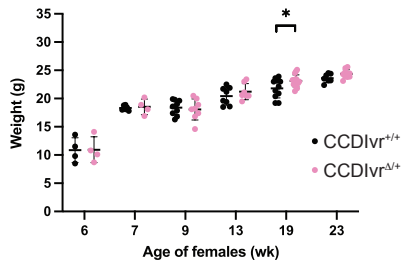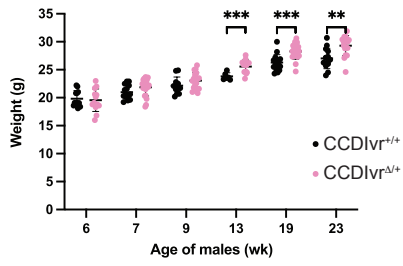

#### Paternal $\Delta$ CCDIvr

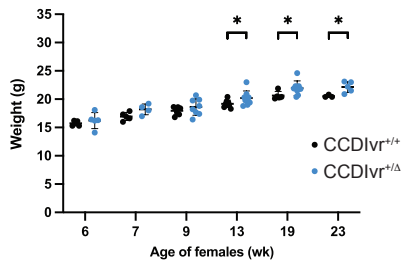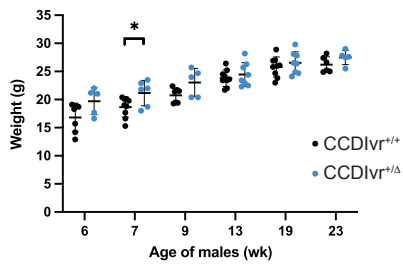

## B

### Adult TA

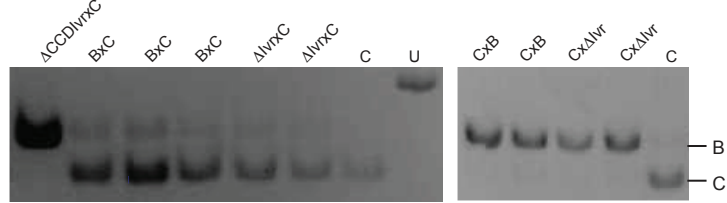

## C

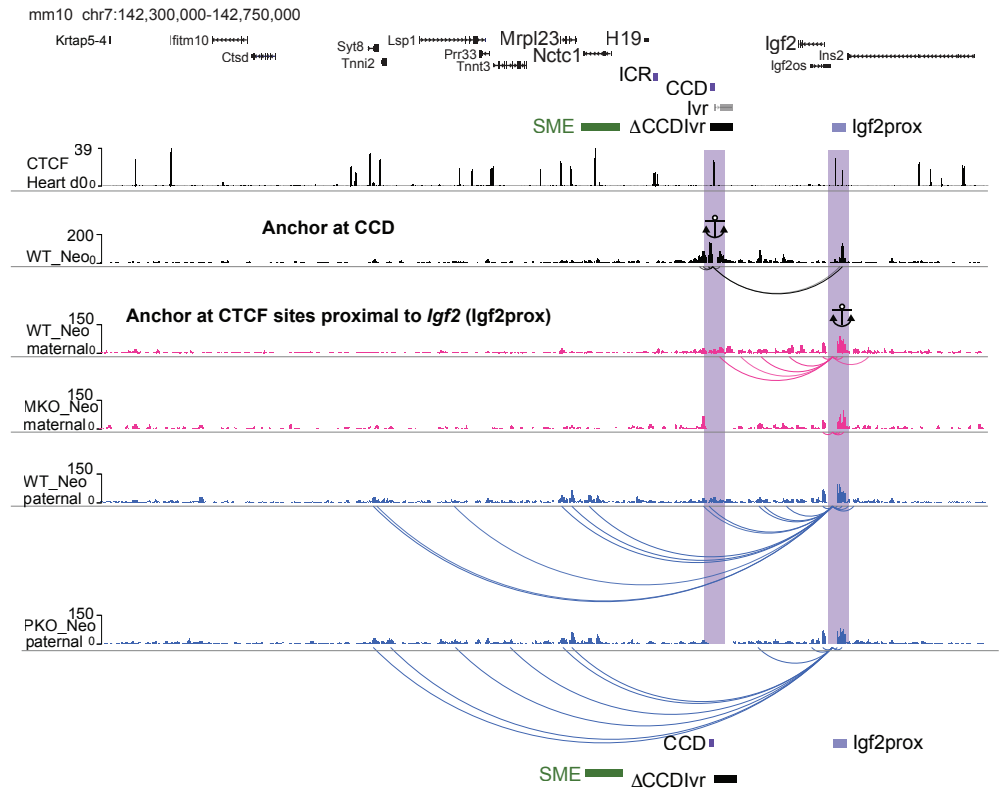

Supplement: S3 Fig — (A) For adult, weights of WT versus mice inheriting the maternal ΔCCDIvr allele (top panels) and WT versus mice inheriting the paternal ΔCCDIvr allele (bottom panels) (Two-tailed Welch’s t-test: *p < 0.05, **p < 0.01, ***p < 0.001; error bars represent SD). (B) Allelic Igf2 expression in adult TA from mice inheriting the ΔIvr allele. Igf2 RT-PCR products were digested with MluCI to discriminate the parental B (WT B, ΔIvr, ΔCCDIvr) and C alleles. (C) and (D) All data is presented on WashU Epigenome Browser mouse mm10: chr7:142,300,000–142,750,000. (C) From top to bottom, the following are depicted: Genes, regulatory elements (SME, ICR, CCD) and probes are annotated (Sheet C in S2 Table). WashU Epigenome Browser CTCF ChIP Seq tracks (Sheet D in S2 Table). Below are normalized Capture-C interaction frequencies of F1 hybrid neonatal SkM from the viewpoint of anchors at CCD and at Igf2prox (Sheet F in S5 Table). CCD and Igf2prox anchored regions are highlighted (purple bars). Black tracks designate interactions from anchor (CCD) without an available polymorphism to discriminate alleles. Pink and blue tracks designate maternal and paternal alleles, respectively, and darker intensities correspond to stronger interactions. The biallelic interaction between sequence adjacent to CCD and Igf2prox occurs on the wild-type allele but is absent from ΔCCDIvr alleles in neonatal SkM. Normalized Capture C tracks to/from Igf2prox and CCD anchors (purple bars) on wild-type (WT, WT_maternal or WT_paternal) and ΔCCDIvr (MKO_maternal and PKO_paternal) alleles in neonatal SkM. Probe and regulatory regions are also annotated below the Capture C data. (PDF) [file pgen.1011834.s003.pdf]

**A**

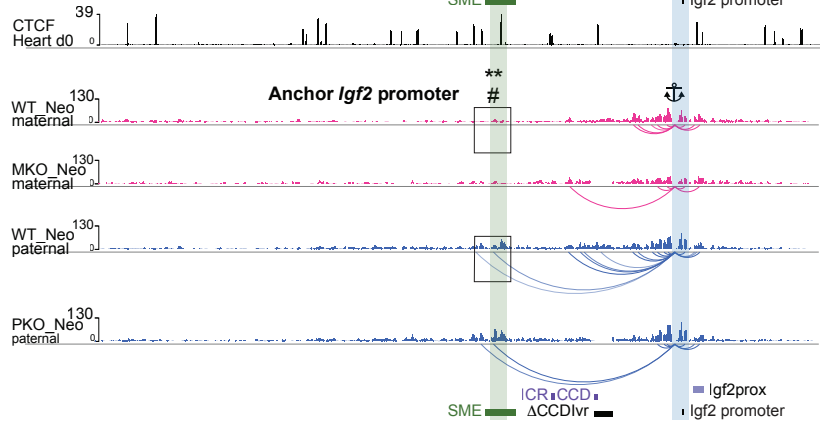

# B

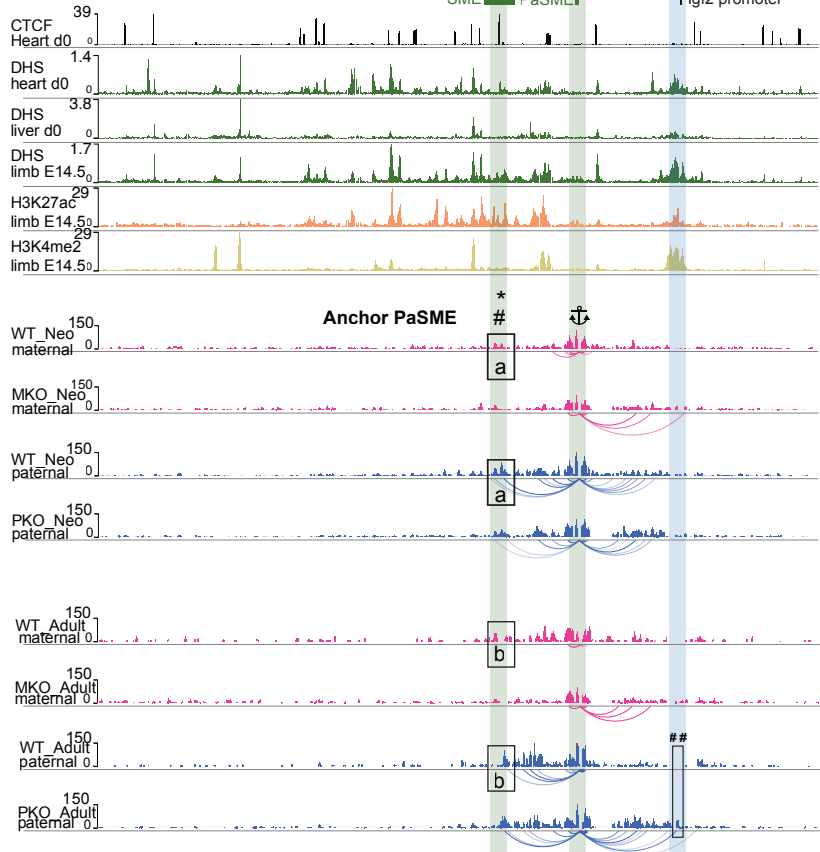

Supplement: S4 Fig — (A) and (B) All data is presented on WashU Epigenome Browser mouse mm10: chr7:142,300,000–142,750,000. Presented from top: annotated genes, regulatory elements and probes and WashU Epigenome Browser CTCF ChIP Seq track (Sheet A and C in S2 Table and Sheet A in S4 Table). Normalized allele-specific Capture-C peaks on WT, MKO and PKO alleles. (A) Captured interactions from anchor at Igf2 promoter. As measured on WT alleles, boxed regions depict paternal specific interaction of Igf2 promoter to Nctc1/SME region (green bar) (Sheet G in Table 5). (B) Analysis of putative adult skeletal muscle specific enhancer (PaSME) region (green bar) in embryonic and neonatal limb (Sheet A in S2 Table, mm10 chr7:142599494–142601493). All data presented on WashU Epigenome Browser mouse mm10 and as referenced in Sheet A in S2 Table. ENCODE DNase-seq (DNase Hypersensitivity Site, DHS) data from neonatal heart and liver and embryonic limb, and H3K27ac and H3K4me2 ChIP-seq peaks in embryonic limb are shown. Below, captured interactions from anchor at PaSME in neonatal and adult skeletal muscle. On WT alleles, boxed regions depict paternal specific interaction to Nctc1/SME region (‘a’ significant, ‘b’ trending, Sheets H and I in S5 Table). On adult PKO allele, a boxed region at Igf2 promoter indicates significant enhancer-promoter (E-P) interactions (Sheet J in S5 Table). SME and PaSME regions are highlighted with green bars and Igf2 promoter region is highlighted with a blue bar. (A) and (B) Wald statistics using two methods to identify peaks are presented (* or # p < 0.05, ** or ## p < 0.01, Sheets G, H and J in S5 Table). (PDF) [file pgen.1011834.s004.pdf]

**A**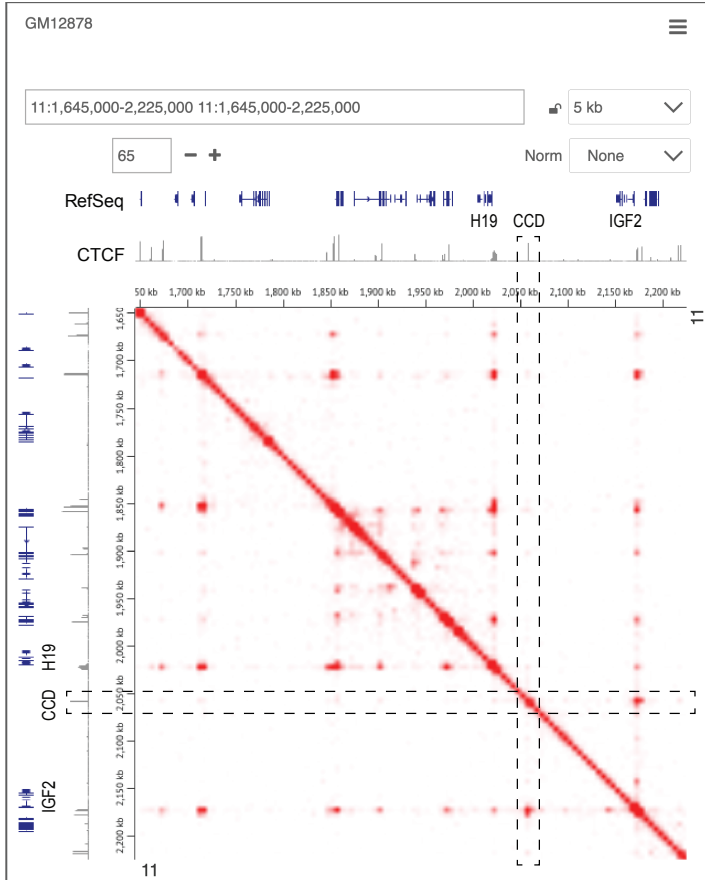**B**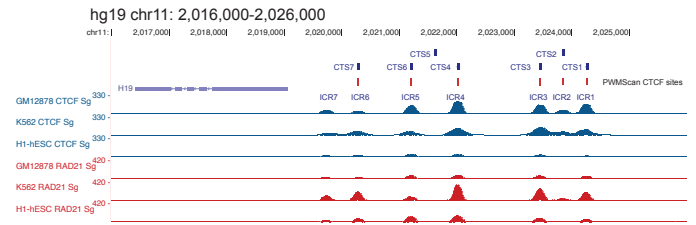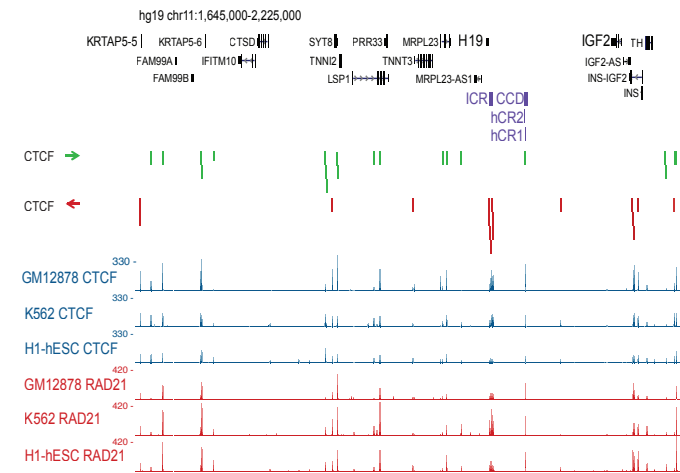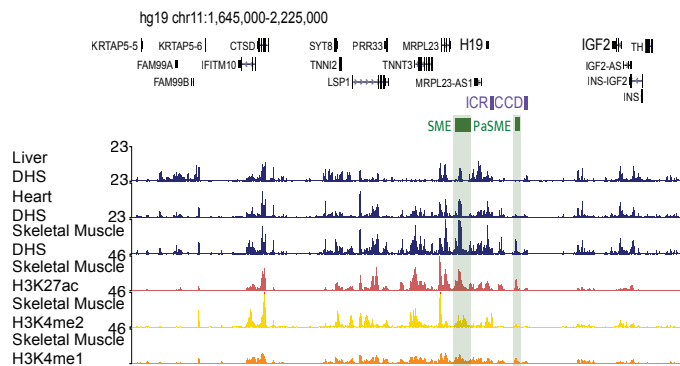

Supplement: S5 Fig — (A) 3D and 2D chromatin structure at human IGF2/H19 locus (hg19 chr11:1,645,000–2,225,000). Source of publicly available data is presented in Sheet B in S2 Table. Juicebox software 3D architecture data [chromatin interaction analysis by paired-end tag sequencing (ChIA-PET)] in human GM12878 cells. The conserved interaction between CCD and proximal Igf2 CTCF sites is presented in dashed boxes. RefSeq Genes, CCD, CTCF ChIP-seq peaks (GM12878 cells, ENCODE) are noted to left and on top of Juicebox interaction map. Below are UCSC genome and WashU browser views of genes within hg19 chr11:1645000–2225000. Location of ICR, CCD, conserved regions hCR1 and hCR2 (upper panel) are designated (Sheet C in S2 Table). CTCF site polarity (green forward arrow/bars for forward, and red reverse arrow/bars for reverse) and CTCF and RAD21 ChIP seq data for indicated cell lines are presented in upper panel (Sheets B and E in S2 Table). In lower UCSC genome browser view, with annotated mouse homologous SME and PaSME regions (green bars), DNase-Seq (DHS) and ChIP-Seq (H3K27ac, H3K4me2, H3K4me1) tracks from designated adult tissues are presented. The Juicebox and UCSC and WashU browser genomes are aligned with each other. (B) CTCF is not bound to originally annotated CTCF binding site (BS) CTS5 in human cell lines; CTCF binding is shown at a newly annotated CTCF BS ICR7 (Sheet E in S2 Table). (PDF) [file pgen.1011834.s005.pdf]
