# Supplementary material for: Igf2 adult-specific skeletal muscle enhancer activity revealed in mice with intergenic CTCF boundary deletion
Source: PLoS Genet. 2025 Aug 29;21(8):e1011834. doi: 10.1371/journal.pgen.1011834 (PMC12416839; doi:10.1371/journal.pgen.1011834)

Fig 1 Gel images - lanes in dashed box are presented in indicated figure.

Fig 1C Li

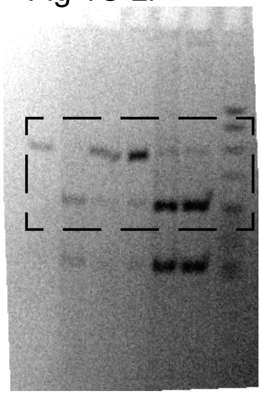

Fig 1D Li

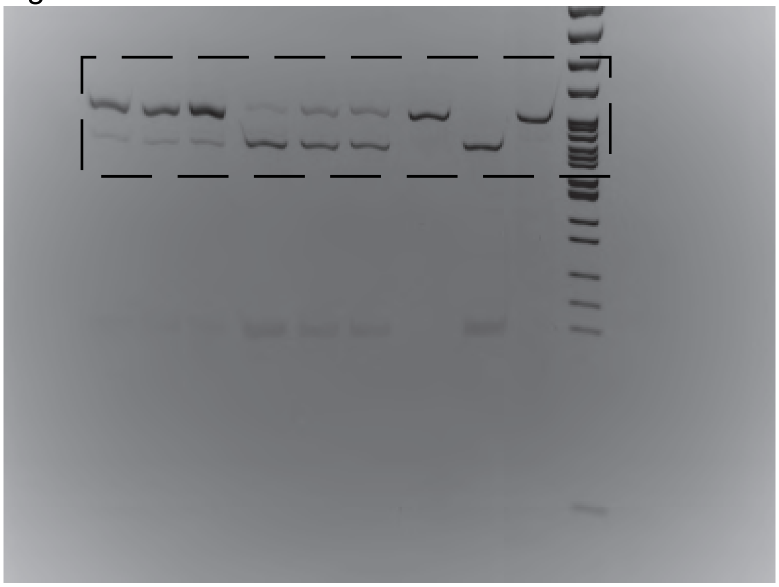

Fig 1D H

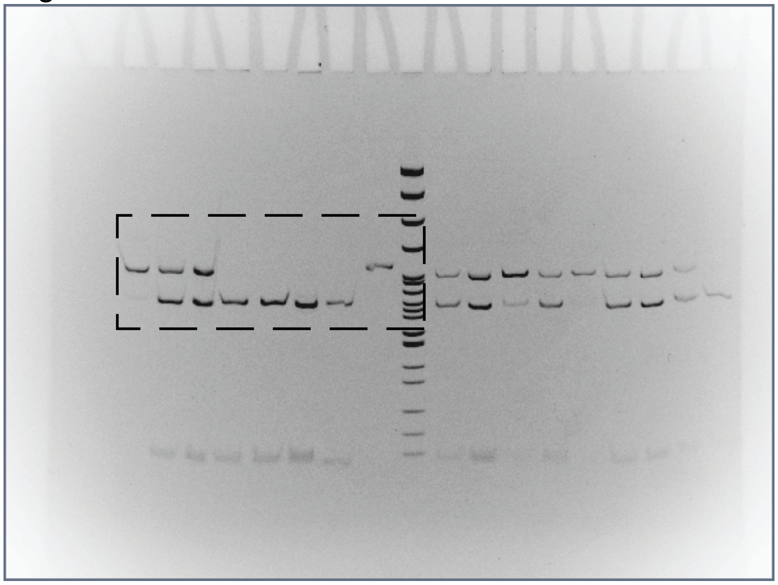

Fig 1D SkM

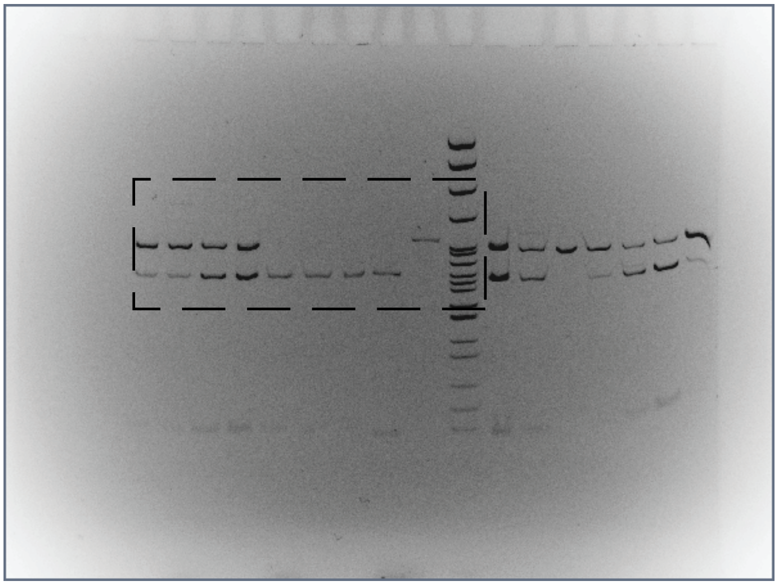

Fig 2 Gel images - lanes in dashed box are presented in indicated figure.

Fig 2A Li

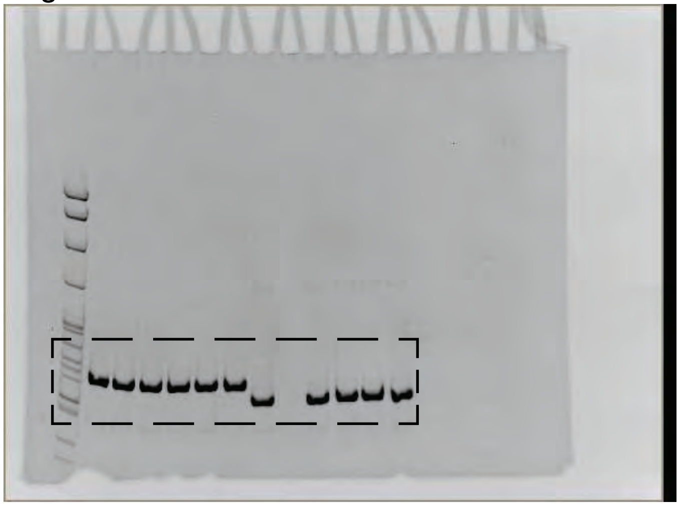

Fig 2A T

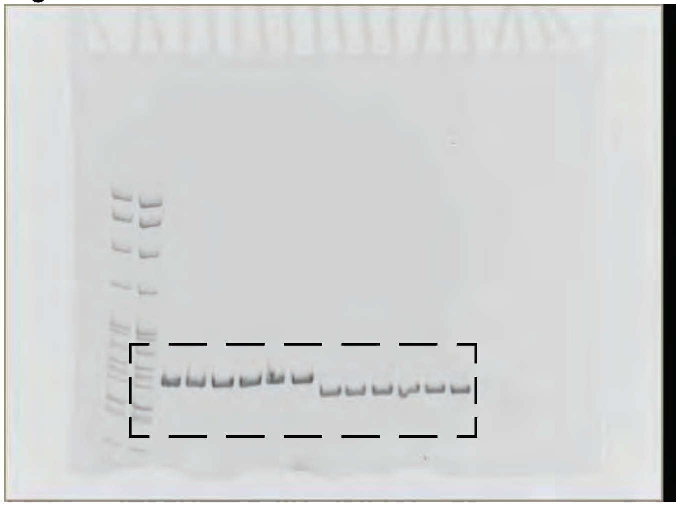

Fig 2B Li

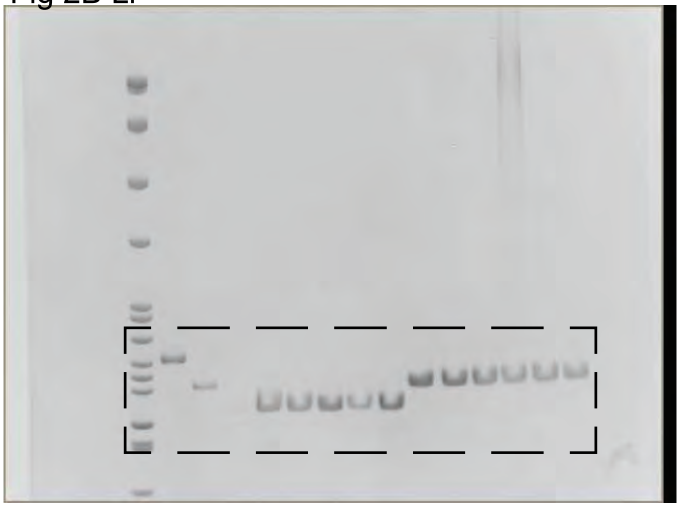

Fig 2B T

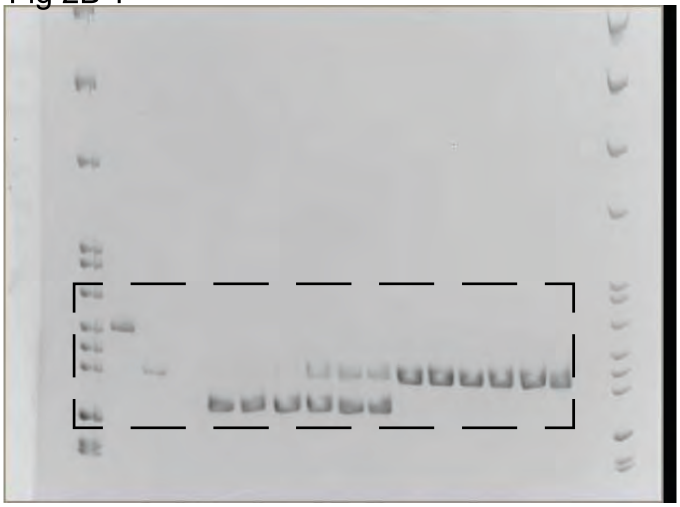

Fig 2B H

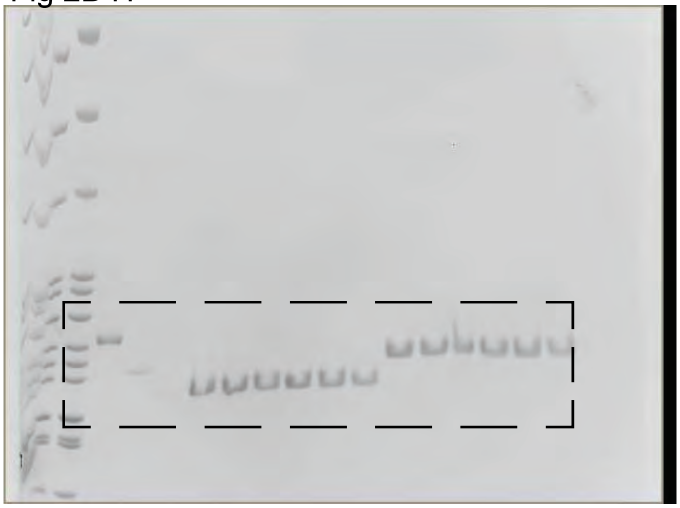

Fig 2D Adult and Neonatal muscle

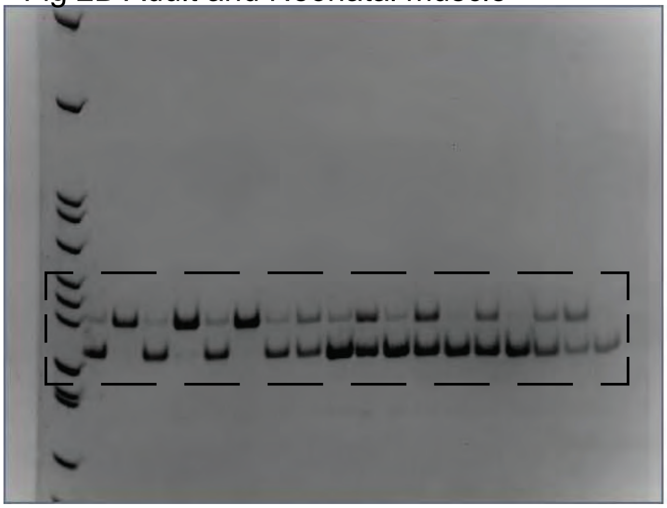

Fig 4 Gel images - lanes in dashed box are presented in indicated figure.

Fig 4C H19 GAS

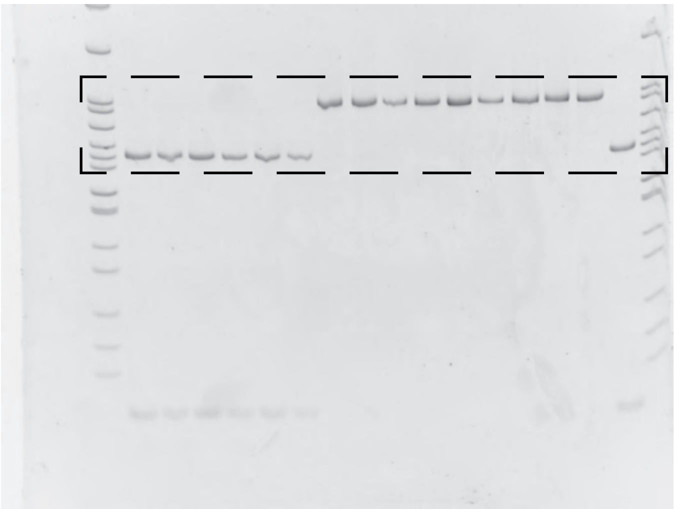

Fig 4D H19 GAS

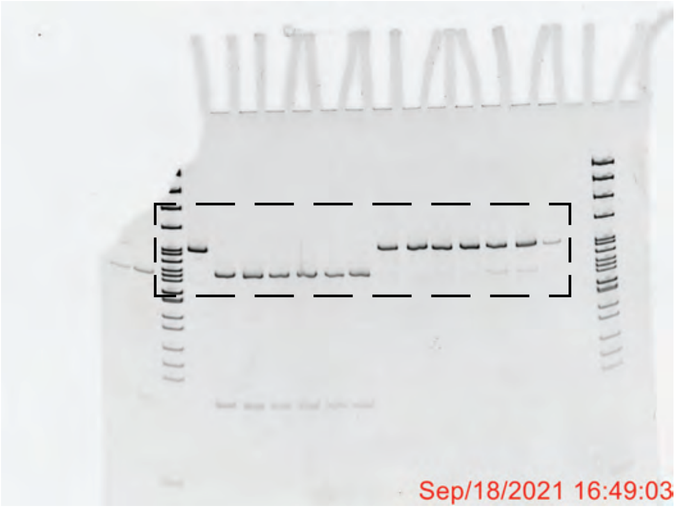

Fig 4E Igf2 GAS

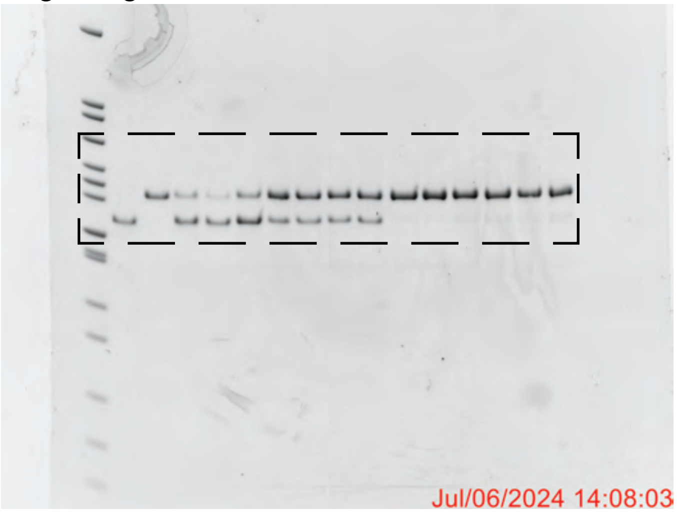

S2B Fig - 3’probe Southorn blot - lanes in dashed box in indicated figure (reverse order).

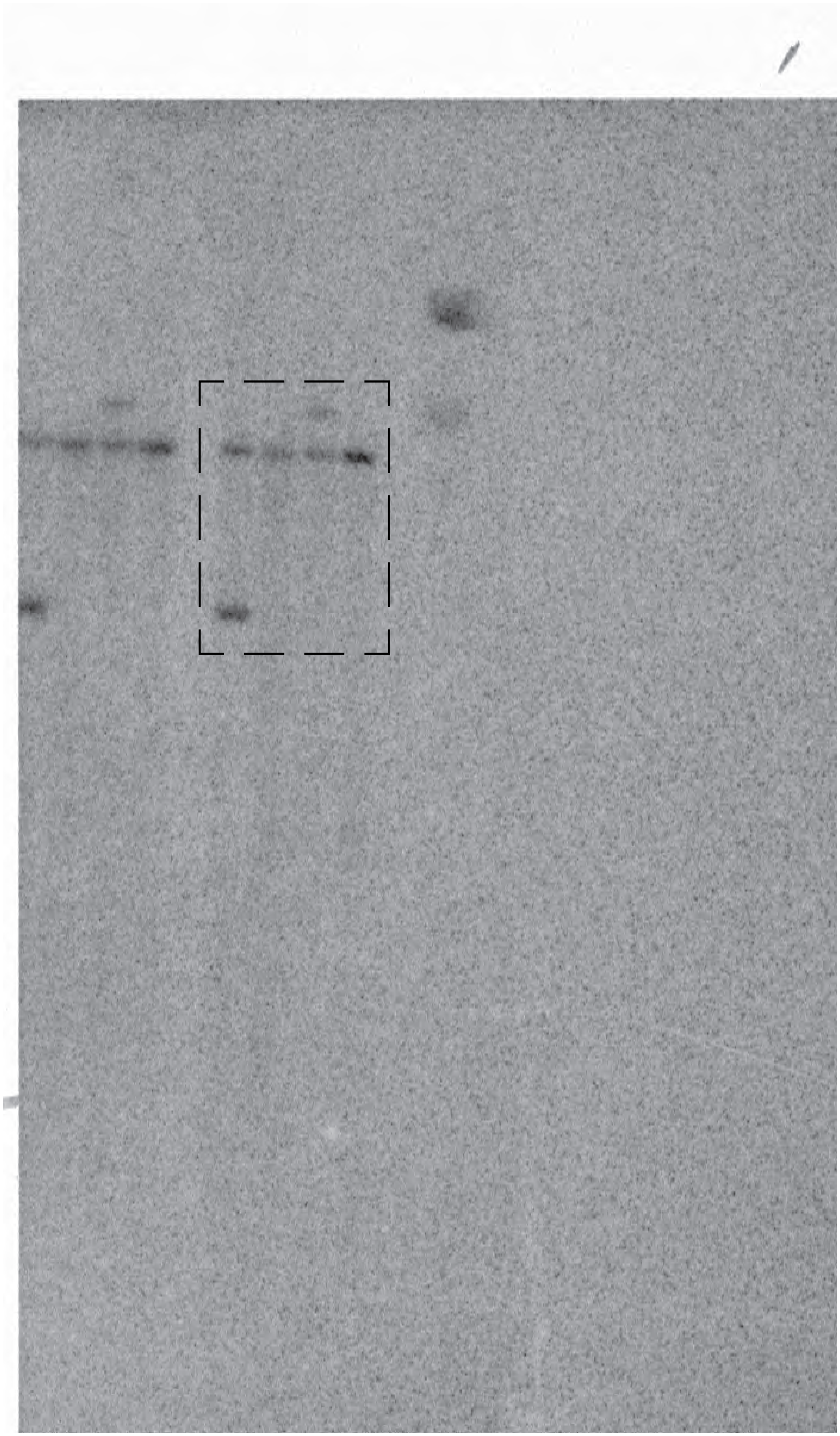

S2B Fig - 5’probe Southorn blot - lanes in dashed box in indicated figure.

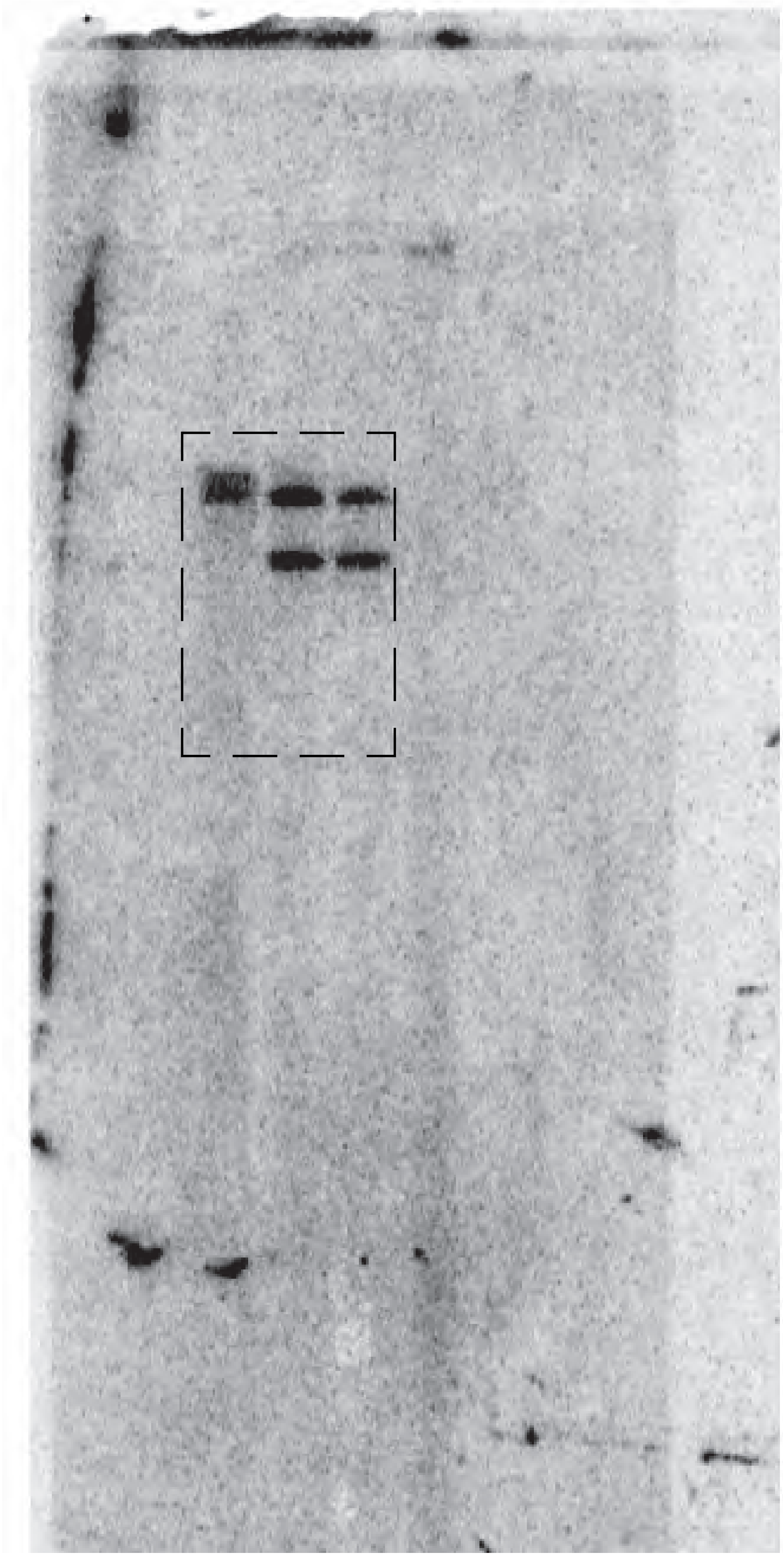

S2D Fig Gel images - lanes in dashed box are presented in indicated figure.

S2D Fig H19 Li

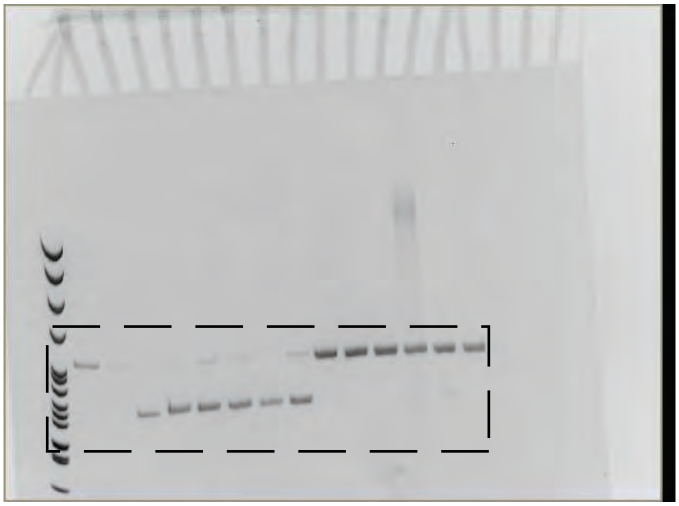

S2D Fig H19 T

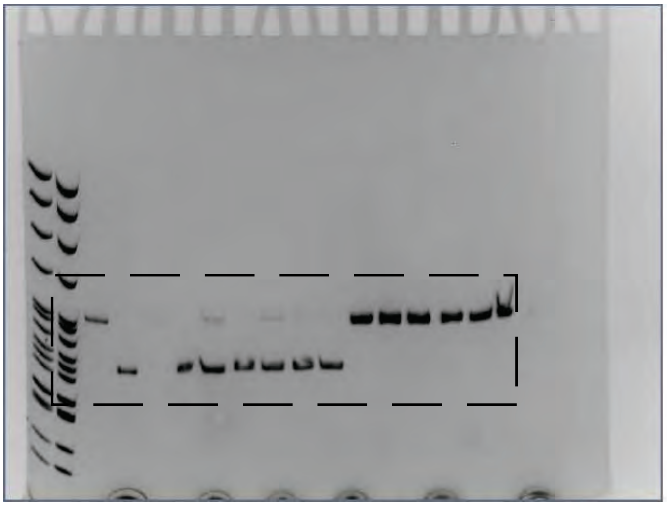

S2D Fig H19 H

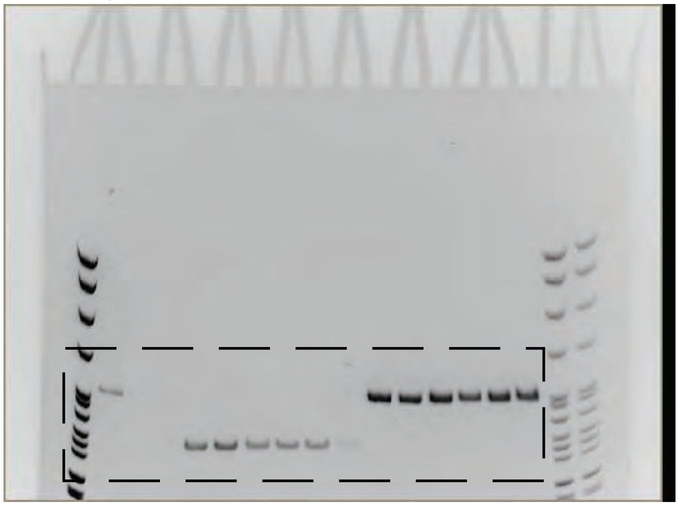

S3B Fig Gel images - lanes in dashed box are presented in indicated figure.

S3B Fig Igf2 Adult TA _BxC and ΔIvrxC samples

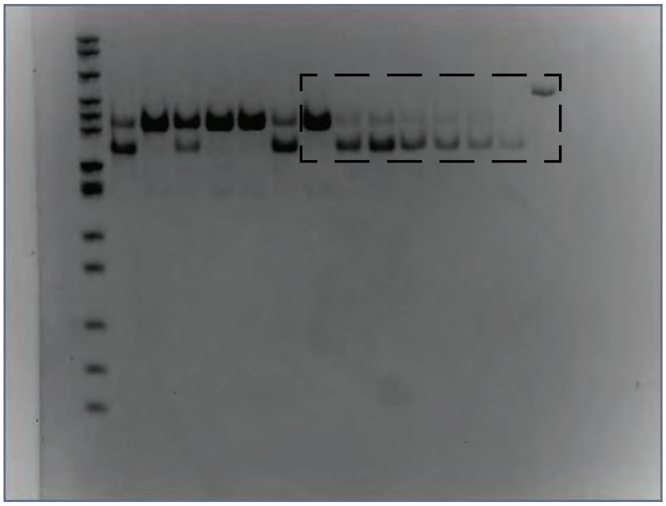

S3B Fig Igf2 Adult TA _CxB and CxΔIvr samples

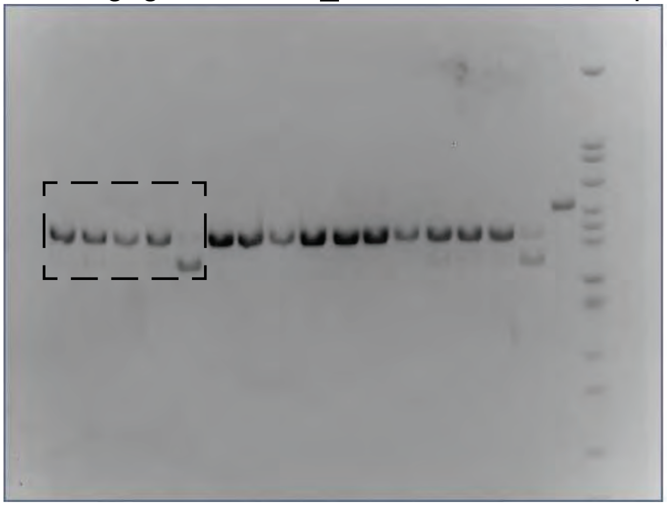

Supplement: S1 Data — (PDF) [file pgen.1011834.s011.pdf]
