# Supplementary material for: Igf2 adult-specific skeletal muscle enhancer activity revealed in mice with intergenic CTCF boundary deletion
Source: PLoS Genet. 2025 Aug 29;21(8):e1011834. doi: 10.1371/journal.pgen.1011834 (PMC12416839; doi:10.1371/journal.pgen.1011834)
Supplement: S2 Fig — (A) UCSC genome browser view of Igf2/H19 locus with mapped CCD, conserved regions CR1 and CR2, DHS, IvrEx1, IvrEx2 and IvrEx3 sequence (Sheet C in S2 Table), above, and targeting vector and targeted loci ΔIvr + neo, ΔIvr (after Flp mediated removal of neo) and ΔCCDIvr (after Cre mediated removal of CCD), below. Rr1em1.1Msb, Rr1em1.2Msb and Rr1em1.2Msb are the MGI approved nomenclature for the respective targeted alleles. The endogenous locus (thick black line) depicts endogenous DraI sites, the sites where DraI and loxP sequences (pink bars) were introduced (insert site), TALEN cut sites (T), CCD (purple box), Ivr transcription unit (gray boxes), targeting vector 5’ and 3’arms (open boxes) and the 5’ and 3’ sequence used for Southern blot analyses (5’P and 3’P, dark gray boxes). In addition to the 5’ and 3’ arm (thick black line), the targeting vector includes introduced DraI site, loxP sites (pink bars), Frt (blue bars) flanked Neo-cassette (open box), DT cassette (open box), and BSSKII (dashed line). Primer pairs (c, d, e, f) used to genotype the targeted loci before and after neo and CCD removal are depicted below the endogenous and target loci. (B) DraI digested DNA analyzed by Southern blots. Using the 5’P probe, the 18.2kb endogenous and 8.9kb targeted DraI fragments are detected. Using the 3’P probe, the 7.2 endogenous and 8.9kb, 7.1kb and 4.1kb target DraI fragments are detected. (C) Total Igf2 expression in CCDIvr+/+ versus CCDIvr+/Δ (paternal ΔCCDIvr) neonatal tongue. (D) Allele-specific H19 expression in neonatal liver (Li), tongue (T) and heart (H) from WT and ΔCCDIvr alleles. H19 qRT-PCR products are digested with Cac8I to discriminate the parental B (WT B, ΔCCDIvr) and C alleles. (E) Total H19 expression in CCDIvr+/+ versus CCDIvrΔ/+ (maternal ΔCCDIvr) neonatal tongue. (F) Weights of WT (CCDIvr+/+) versus maternal deletion (CCDIvrΔ/+) and WT (CCDIvr+/+) and paternal deletion (CCDIvr+/Δ) neonatal littermates. No statistical difference between weights of [file pgen.1011834.s002.pdf]

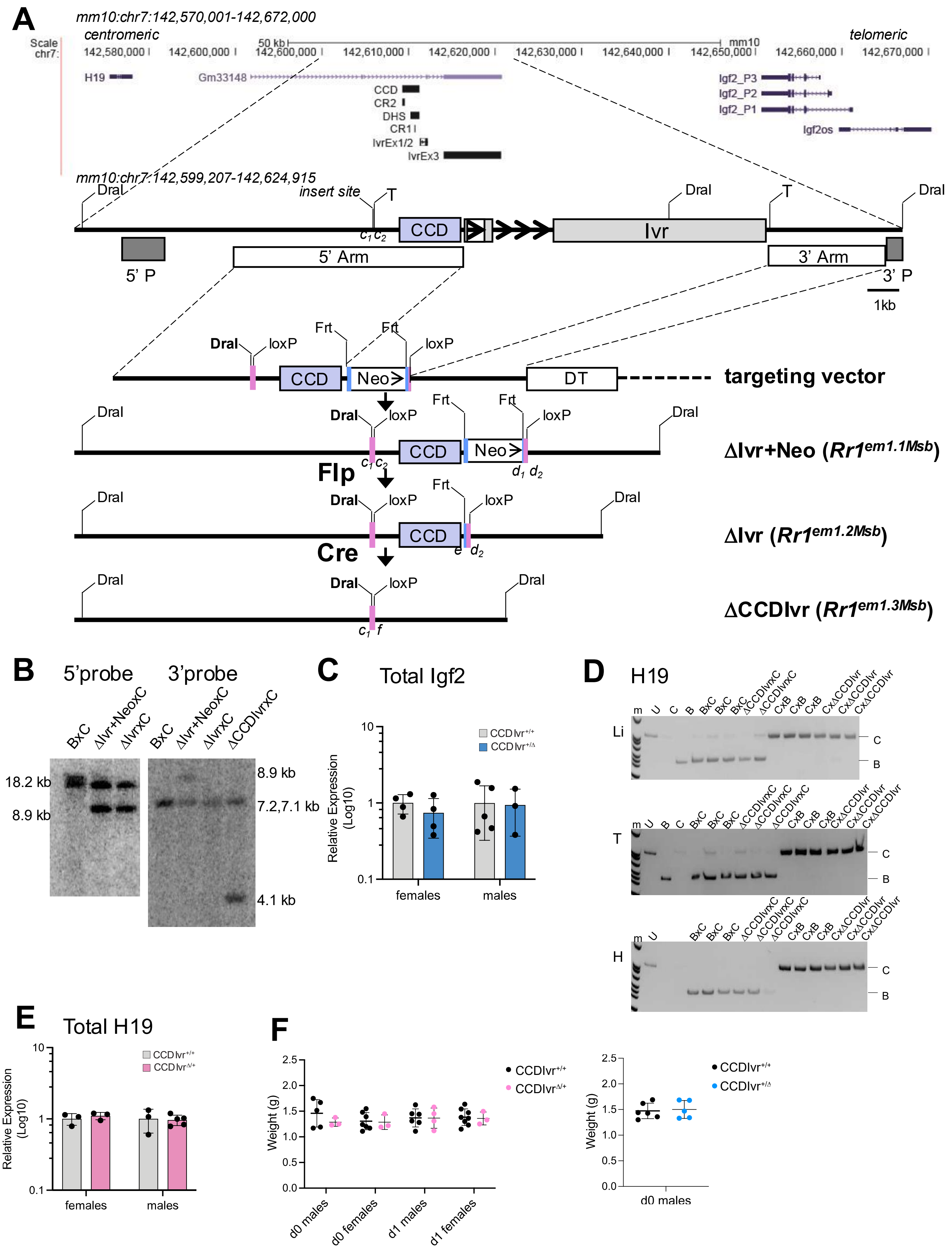
A
mm10:chr7:142,570,001-142,672,000
centromeric
telomeric
Scale
chr7:
50 kb
mm10
142,580,000
142,600,000
142,600,000
142,610,000
142,620,000
142,630,000
142,640,000
142,650,000
142,660,000
142,670,000
H19
Gm33148
CCD
CR2
DHS
CR1
IvrEx1/2
IvrEx3
Igf2_P3
Igf2_P2
Igf2_P1
Igf2os
mm10:chr7:142,599,207-142,624,915
DraI
insert site
T
CCD
Ivr
3' Arm
5' Arm
5' P
3' P
1kb
Frt
loxP
Neo
DT
targeting vector
ΔIvr+Neo (Rr1em1.1Msb)
Flp
ΔIvr (Rr1em1.2Msb)
Cre
ΔCCDIvr (Rr1em1.3Msb)
B
5'probe
3'probe
BxC
ΔIvr+NeoxC
ΔIvrxC
ΔCCDIvrxC
18.2 kb
8.9 kb
7.2,7.1 kb
4.1 kb
C
Total Igf2
Relative Expression (Log10)
CCDIvr+/+
CCDIvr+/Δ
females
males
D
H19
m
U
C
B
BxC
CxB
CxΔCCDIvr
Li
T
H
E
Total H19
CCDIvrΔ/+
F
Weight (g)
d0 males
d0 females
d1 males
d1 females
